# Overexpression of GmUBC9 Gene Enhances Plant Drought Resistance and Affects Flowering Time *via* Histone H2B Monoubiquitination

**DOI:** 10.3389/fpls.2020.555794

**Published:** 2020-09-04

**Authors:** Kai Chen, Wen-Si Tang, Yong-Bin Zhou, Zhao-Shi Xu, Jun Chen, You-Zhi Ma, Ming Chen, Hai-Yan Li

**Affiliations:** ^1^ College of Life Sciences, Jilin Agricultural University, Changchun, China; ^2^ Institute of Crop Science, Chinese Academy of Agricultural Sciences (CAAS)/National Key Facility for Crop Gene Resources and Genetic Improvement, Key Laboratory of Biology and Genetic Improvement of Triticeae Crops, Ministry of Agriculture, Beijing, China

**Keywords:** soybean, ubiquitin-conjugating enzyme, histone monoubiquitination, drought tolerance, regulation of flowering

## Abstract

Ubiquitylation is a form of post-translational modification of proteins that can alter localization, functionality, degradation, or transcriptional activity within a cell. E2 ubiquitin-conjugating enzyme (UBC) and E3 ubiquitin ligases are the primary determinants of substrate specificity in the context of ubiquitin conjugation. Multiubiquitination modifies target proteins for 26S proteasome degradation, while monoubiquitination controls protein activation and localization. At present, research on the monoubiquitination, especially histone monoubiquitination, has mostly focused on model plants with relatively few on crop species. In this study, we identified 91 *UBC*-like genes in soybean. The chromosomal localization, phylogenetic relationships, gene structures, and putative cis-acting elements were evaluated. Furthermore, the tissue-specific expression patterns of *UBC* Class I genes under drought stress were also investigated. Among Class I genes, *GmUBC9* induction in response to drought stress was evident, and so this gene was selected for further analysis. GmUBC9 localized to the nucleus and endoplasmic reticulum. The overexpression of *GmUBC9* in *Arabidopsis* led to enhanced tolerance for drought conditions across a range of stages of development, while overexpression in soybean hairy roots similarly led to improvements in tolerance for drought conditions, increased proline content, and reduced MDA content in soybean seedlings compared to wild type plants. HISTONE MONOUBIQUITINATION 2 (HUB2), an E3-like protein involved in histone H2B ubiquitylation (H2Bub1), was found to interact with GmUBC9 through Y2H analysis and BiFC assays in *Arabidopsis* and soybean. Under drought conditions, the level of H2Bub1 increased, and transcription of drought response genes was activated in *GmUBC9* transgenic *Arabidopsis* and soybean. In addition, *GmUBC9* transgenic *Arabidopsis* and soybean showed a late-flowering phenotype and had increased expression levels of the flowering related genes *FLC* and *MAF4*. These findings indicate that GmUBC9 is important for drought stress response and regulation of flowering time in soybean.

## Introduction

Post-translational protein modification plays a very important role in living organisms. It makes proteins more complex in structure, more complete in function, more precise in regulation and more specific in function (Cruz et al., 2019). Common post-translational modifications of proteins include ubiquitination, phosphorylation, glycosylation, methylation, acetylation and so on (Wang and Wang, 2019). Ubiquitination is an important post-translational modification of proteins. It plays a very important role in the growth and development of eukaryotes and their response to adverse environments. Protein ubiquitination can regulate life span or abnormally folded protein stability, change of protein subcellular localization, protein assembly, and protein activity, etc. (Vierstra, 2012). In Arabidopsis thaliana, the protein involved in this pathway accounted for about 5% of the total protein, ubiquitination can be seen playing a very broad and important role during plant growth (Vierstra, 2003; Smalle and Vierstra, 2004).

According to the different length of the ubiquitin substrate and the ubiquitin chain, ubiquitin can be divided into monoubiquitin, polyubiquitin, and polyubiquitin. If the target protein binds to a single ubiquitin molecule, it is called mono-ubiquitination. Multiple Lys residues of target protein are simultaneously labeled by a single ubiquitin molecule, known as multi-ubiquitination. While single Lys residues of target proteins are labeled by multiple ubiquitin molecules. It is called poly-ubiquitination (Passmore and Barford, 2004). Protein polyubiquitination is a common modification regulation phenomenon in biology, which is usually a signal of protein degradation, and a few can also regulate the activity of proteins and the interaction between proteins. Similar to reversible modifications in polyubiquitination (Passmore and Barford, 2004; Kraft et al., 2005), mono-ubiquitination is a reversible regulatory process in which a single ubiquitin molecule is linked to a target protein by forming a heterogeneous bond between its Gly76 and Lys inside the target protein (Passmore and Barford, 2004). After mono-ubiquitination, the subcellular localization, structural activity and function of target proteins are changed, and their interactions with other proteins are affected. In general, monoubiquitinated target proteins have the functions of regulating transcriptional activation and extension and gene expression (Haglund et al., 2003; Hicke and Dunn, 2003; Weake and Workman, 2008). Unlike polyubiquitin, monoubiquitinated proteins are usually stable and hard to degrade (Pavri et al., 2006; Shilatifard, 2006). The most common form of protein mono-ubiquitination is histone mono-ubiquitination, the most typical example being histone H2A (Wang and Shen, 2018) and H2B (Weake and Workman, 2008). As the physiological template carrying genetic information, chromatin contains genomic DNA packaged by evolutionarily conserved proteins: histones H1, H2A, H2B, H3 and H4 (Zhou et al., 2015; Wang and Shen, 2018). The heterodimers formed by histones H2A and H2B are outside the nucleosome and are therefore more susceptible to be covalent modified (Luger et al., 1997). Among them, histone H2B monoubiquitination is a hot epigenetic modification recently studied. Monoubiquitination can profoundly impact plant growth and development processes including embryogenesis, organogenesis, leaf senescence, phytohormone and light response, and plant defense response (Zhou et al., 2017; Sun et al., 2020; Zhao et al., 2020), which is closely related to the enzymes needed in the process of histone H2B monoubiquitination.

No matter the protein mono-ubiquitination, multi-ubiquitination or poly-ubiquitination is completed by the ubiquitination modification pathway, which involves ubiquitin molecules, ubiquitin-activating enzyme E1, ubiquitin-binding enzyme E2 and ubiquitin-ligase E3 (Pickart, 2001; Glickman and Ciechanover, 2002). In *Arabidopsis*, a total of 37 E2-like proteins have been predicted to exist based on the fact that they encode putative UBC domains with a cysteine in the active site (Kraft et al., 2005), and among those E2-like proteins, ubiquitin conjugating enzyme activity had been detected in 17 E2-like proteins, including AtUBC1 and AtUBC2 (Sullivan and Vierstra, 1993; Kraft et al., 2005; Wen et al., 2006). In soybean, sequence analysis eventually determined 91 genes encoding typical UBC domain-containing proteins. The phylogenetic analysis indicated that, of the 91 genes, 71 encode ubiquitin E2 proteins, 11 encode UEV proteins, two encode RUB E2 proteins (RCE), four encode putative SUMO E2, one encodes ELC and two encode UFC1 E2 proteins (Zhang et al., 2018). Expression of AtUBC2 is capable of complementing the UV sensitivity of mutant *rad6* (a E2-like protein) yeast (Zwirn et al., 1997). In yeast, radiation sensitivity of RAD6 functions along with E3-like protein UBR1 to govern N-end rule degradation (Madura et al., 1993), with E3-like protein RAD18 to mediate PCNA monoubiquitination (Hoege et al., 2002), and with the E3-like protein BRE1 to modulate histone H2B monoubiquitination (Sun and Allis, 2002; Hwang et al., 2003; Wood et al., 2003).

HISTONE MONOUBIQUITINATION1/2 (HUB1/2) as an unconventional ubiquitin E3 ligase that is not involved in protein degradation but in the histone H2B modification that is implicated in transcriptional activation in plants (Himanen et al., 2012). HUB1/2-mediated regulation of gene expression plays an important role in plant life such as various developmental processes (i.e. branching, leaf venation pattern, silique development) (Lolas et al., 2010), photomorphogenesis (Bourbousse et al., 2012), regulation of circadian clock genes (Himanen et al., 2012), leaf cuticle formation (Ménard et al., 2014), flowering time control (Gu et al., 2009; Zhao et al., 2019) and defense responses (Dhawan et al., 2009; Zou et al., 2014). H2Bub1 can impact histone methylation including H3K4me3 (Chen et al., 2019; Fiorucci et al., 2019) as well as H3K36me3 (Cao et al., 2008; Zhao et al., 2019), which regulate flowering time through modulating the expression of the floral repressor *FLOWERING LOCUS C* (*FLC*). H2Bub1 is centrally involved in plant drought responses and in abscisic acid (ABA) signaling as exemplified through two E3-like proteins: OsHUB2 and AtHUB2, respectively (Cao et al., 2015; Ma et al., 2019; Chen et al., 2019). At present, efforts toward understanding monoubiquitination, especially histone monoubiquitination, have made considerable progress in some other plants, such as tomato (Zhang et al., 2015) and cotton (Chen et al., 2019). The other thing that has to be mentioned is that HUB1 and HUB2 act non-redundantly and possibly as a hetero‐tetramer in H2B monoubiquitination, whereas UBC1 and UBC2, but not UBC3, act redundantly as E2 in *Arabidopsis* (Fleury et al., 2007; Liu et al., 2007; Cao et al., 2008; Xu et al., 2009).

In soybean, *GmUBC2* is a yeast *RAD6* homolog, the expression of which is detected in all tissues and enhanced in response to salt or drought stress conditions (Zhou et al., 2010), but the functions and regulation mechanism of *GmUBC2* are unknown. The mung bean E2-buquitin conjugating enzyme VrUBC1 is known to positively regulate tolerance to osmotic stressors, potentially through interactions with AtVBP1 (a C3HC4-type E3 ligase) in the context of *Arabidopsis* ABA-driven responses to osmotic stress (Chung et al., 2013). However, how E2 proteins from the leguminous plants positively regulate abiotic stress through ubiquitination is still unknown. In this work, we systematically analyzed all E2 family members of soybean. A total of 91 E2 protein members were identified, and Class I E2 protein member expression patterns in response to a range of stressors were evaluated. We found that a drought responsive E2 member, *GmUBC9*, can confer transgenic soybean tolerance to drought stress and affect flowering time through interaction with the E3-like protein HUB2 and regulation of H2Bub1. Together, our data provides a new understanding of the mechanism of abiotic stress response *via* monoubiquitination in soybean.

## Materials and Methods

### Soybean UBC Family Member Identification

*Arabidopsis* UBC sequences were initially downloaded from the TAIR database (https://www.arabidopsis.org/index.jsp), after which the Phytozome V10.3 (http://phytozome.jgi.doe.gov) soybean genomic database was searched to identify putative UBCs using BLASTP (Sathuvalli et al., 2017; Zhao et al., 2018; Guan et al., 2019). Redundant sequences were eliminated, and Pfam (http://pfam.xfam.org/) was used to confirm the identities of putative UBCs. In addition, ExPASy (https://web.expasy.org/compute_pi/) was used to assess each of these putative UBCs in order to establish their predicted molecular weight (Mw) and isoelectric point (pI) values.

### Chromosome Locations, Phylogenetic Analysis and Motif Elicitation of Soybean *UBC* Genes and UBC Proteins

Culstal X 2.0 (Thompson et al., 1997) was used to align *Arabidopsis* and Fabidae UBC sequences prior to importation into MEGA6.0 (Tamura et al., 2007), which utilized a neighbor-joining approach (1,000 bootstrap repeats) to generate a phylogenetic tree as in prior studies (Zhang et al., 2017). The Phytozome database was referenced in order to establish soybean UBC gene location details.

As for WGD/segmental duplications, MakeBlastDB module of the software NCBI blast+ 2.7.1 (ftp://ftp.ncbi.nlm.nih.gov/blast/executables/blast+/LATEST/) was used for protein sequence library construction and *GmUBC* gene sequence comparison using blastP module. Default parameters were used for all parameters. MCScanX software (http://chibba.pgml.uga.edu/mcscan2/) was used for a collinearity analysis the following parameters settings: MATCH_SCORE, final score=MATCH_SCORE+NUM_GAPS*GAP_PENALTY (default: 2); GAP_PENALTY, gap penalty (default: 10); MATCH_SIZE, number of genes required to call a collinear block (default: 1); alignment significance: E_VALUE, alignment significance (default: 1e-05); MAX_GAPS, maximum gaps allowed (default: 100); OVERLAP_WINDOW, maximum distance (# of genes) to collapse BLAST matches (default: 10) and Circos (http://circos.ca/) was used for visualization (Krzywinski et al., 2009). As for motif elicitation, we used MEME (http://meme-suite.org) version 5.1.1 (Release date: Wed Jan 29 15:00:42 2020 -0800).

### Assessment of Gene Structure and Cis-Acting Elements

The Gene Structure Display Server (GSDS) (http://gsds.cbi.pku.edu.cn/) was used as a tool for assessing exon and intron structures within soybean UBCs, and as a means of detecting conserved domains within these putative proteins. We additionally identified cis-acting elements within a 2kb region upstream of the translation initiation site of each of these UBCs (as determined using the Phytozome database) through the use of the PlantCARE database (http://bioinformatics.psb.ugent.be/webtools/plantcare/html/).

### Tissue-Specific Expression Pattern Analysis of Soybean *UBC* Genes

Transcriptomic data from the Phytozome database was used as a means of assessing the patterns of UBC gene expression within flowers, stems, seeds, leaves, nodules, root hairs, and roots of soybean plants, with heatmaps being generated using the HemI software (Li et al., 2019).

### Real-Time Quantitative PCR (RT-qPCR)

The drought-response pattern sample treatment of soybean UBC Genes was performed as follows (Du et al., 2018), with minor modifications. Specifically, 7 to 10-day-old seedlings were grown in sectors (two plants per sector) of 8 × 8 × 10 cm (L ×W× H) plastic pots. Plants were incubated in a chamber under short-day conditions [8-h light (21°C)/16-h dark (18°C) cycle (light intensity of 110–130 µmol m^−2^ s^−1^)]. Seedlings were fertilized regularly. After two weeks, pots were then subjected to drought conditions and sampling on time (0, 1, 2, 4, 8, 12, and 24 h). During this period (0–24 h), we maintained normal light conditions. RNAiso Plus (Takara, Japan) was utilized to extract total RNA, after which a PrimeScript II first Strand cDNA Synthesis Kit (Takara) was used based on provided directions to prepare cDNA. An ABI7500 instrument (ABI, USA) was then utilized for all RT-qPCR reactions together with the TransStart Top Green qPCR SuperMix (Transgen, China). Data analyses were conducted as in prior reports (Liu et al., 2013), with normalization controls for these analyses including soybean *CYP2* (Glyma12g02790) and *Arabidopsis* *ACTIN2* (At3g18780). All samples were assessed in triplicate with the primers compiled in **Supplemental Table S4**. Three biological replicates were applied in the experiment.

### Intracellular Localization Analyses

In order to evaluate *GmUBC9* localization within cells, we utilized a vector encoding GFP under the control of the cauliflower mosaic virus (CaMV) 35S promoter as well as the *GmUBC9* endogenous promoter, with a stop codon-free version of the *GmUBC9* coding sequence being inserted proximal to the GFP N-terminal sequence. *Arabidopsis* protoplasts were then transformed with this vector as in prior analyses (Cui et al., 2019). Protoplasts were incubated for 18–24 h at 23°C, after which they were examined *via* confocal laser scanning microscopy (CLSM; Carl Zeiss LSM 700, Germany). The excitation of GFP and RFP were conducted at 488 and 561 nm, respectively, with emission being captured between 495-540 nm and 580–620 nm, respectively. See **Supplementary Table S4** for primer-specific sequences details.

### Transgenic *Arabidopsis* Plant Preparation

The *GmUBC9* ORF was first subject to amplification prior to cloning into a pCAMBIA1302 vector under the control of its endogenous promoter. The pCAMBIA1302-*GmUBC9* vector was then utilized to transform the *Agrobacterium tumefaciens* GV3101 strain, which was then used to transform *Arabidopsis* Col-0 plants *via* a floral dipping strategy (Clough and Bent, 1998). Hygromycin (40 mg/L) was then used to assess collected seeds in order to identify resistance, with RT-qPCR being used to confirm the identities of T3-transformed plants based upon *GmUBC9* expression levels. The RT-qPCR analysis was performed with 3 independent technical replicates of each transformed lines.

### Soybean Hairy Root Transformation Using *Agrobacterium rhizogenes*

A pCAMBIA3301-*GmUBC9* overexpression vector was prepared by amplifying and cloning the *GmUBC9* ORF (under the control of its endogenous 2.0kb upstream promoter region) into the pCAMBIA3301 vector, with an empty version of this vector serving as a control. A highly efficient *A. rhizogenes*-mediated transformation approach was utilized to introduce these vectors into soybean hairy roots as in past studies (Li et al., 2019). The transformed roots were confirmed by gene cloning and qRT-PCR analysis (the analysis was performed with 3 independent technical replicates of each root lines) (**Supplementary Figure S5**).

### Drought Tolerance Analyses

We utilized 1/2 Murashige and Skoog (MS) medium as a means of growing both transgenic and wild-type (WT) *Arabidopsis* lines at 22°C, with a 16-hour light/8-hour dark photoperiod. For root length analyses, we transferred 7-day-old uniformly-germinated seeds onto 10% PEG6000-supplemented 1/2 MS medium. Three biological replicates were applied in the experiments. Tolerance for drought stress was assessed *via* withholding water from 3-week-old plants for a 4-day period, followed by 2 days of re-watering in soil. The method of watering is to pour 40ml deionized water into each pot at 4:00 PM every day. There were three biological replications in this experiment.

For soybean, the *GmUBC9*-OE and CK seedlings that exhibited hairy roots between 3 and 5 cm in lengths were planted into pots that contained a 1:1 mixture of humus/vermiculite, after which they were allowed to grow for 6–8 days in a greenhouse. Next, drought was simulated by not watering these plants for 6days, after which they were re-watered for a 3-day period. Watering consisted of pouring 80 ml deionized water into each pot at 4:00 PM every day. Four biological replicates were applied in the experiments (n=4).

### Main Length, Surface Area and Fresh Weight Analyses

An Expression 11000XL root system scanning analyzer (Epson, Nagano, Japan) fitted with a transparency adapter and calibrated by Regent Instruments, Inc. was used to scan roots. Each root was arranged on the scanner bed so that there was as much separation as possible between secondary roots with a minimum of overlap. After each root scan, the image was analyzed for length, area, and volume. The root system WinRHIZO 2016 program (Regent Instruments, Inc., Quebec, Canada) was used to analyze root data (Kraft and Boge, 2001).

The WinRHIZO root analysis system uses a high-quality graphics scanner to obtain high-resolution color or black and white images of plant roots. The scanner is equipped with a special dual-light lighting system under the scanning panel and the upper cover, and a dual-light calibration area is reserved on the scanning panel. In addition, there are special, high transparency root trays of different sizes. During the scanning, the light source under the panel and the light source in the upper cover plate are simultaneously scanned the root samples in the over transparency root plate, which can avoid the influence of shadow and non-uniformity easily generated during root scanning and effectively ensure the acquired image quality.

WinRHIZO can read images in standard formats such as TIFF and JPEG. For the obtained images, the software for insertion and decryption was used, and meanwhile, the scanner.cal calibration file configured by the manufacturer for the Scanner was used to analyze the high-quality root images obtained by scanning. The root length, diameter, area, volume, root tip and other basic morphological parameters were measured and calculated.

A BSA224S-CW 1/10,000 analytical balance (Sartorius, Beijing, China) was used to quantify root fresh weight. Four biological replicates were applied in the experiments (n=4).

### MDA and Proline Content Analyses

For metabolite measurements, the leaves of drought-stressed CK (control) plants and *GmUBC9*-OE plants were collected to assess proline and MDA contents. Briefly, drought stress was induced as above (6–8 days with normal watering, followed by the withholding of water for 6 days), after which *GmUBC9*-OE and CK seedlings were collected. A previously described approach (Du et al., 2018; Zhang et al., 2019) was used to assess MDA levels. Briefly, ~0.1 g of leaf tissue from four plants were collected, with optical density (OD) at 532 and 600 nm being recorded and used to calculate MDA levels as follows: MDA content (nmol/g FW) = 51.6×(OD_532_–OD_600_)/0.1. A previously described approach (http://www.cominbio.com/uploads/soft/180727/1-1PHGF414.pdf) was also used to assess proline levels, with OD values at 520 nm for ~0.1 g leaf samples from four plants being determined and used to assess proline content as follows: proline content (g/g FW) = 38.4×(OD_520_ + 0.0021)/0.1. A Varioskan LUX Multimode Microplate Reader (Thermo Fisher Scientific, USA) was used for measuring OD values. Three samples of a single transgenic line (n=4) of the same genotype were sampled at the same location (Zhang et al., 2019).

### DAB Staining

Drought stress was induced as above (6–8 days with normal watering, followed by the withholding of water for 6 days), after which *GmUBC9*-OE and CK plants were harvested for DAB staining. A DAB solution (Solarbio, Beijing, China) was used to soak collected leaf samples for 14 h, after which they were added to 75% ethanol for de-coloration until white (Du et al., 2018).

### Yeast Two-Hybrid Assays

All tested genes were cloned into the pGBKT7 and pGADT7 vectors, which were subsequently co-transformed into AH109 yeast. The methods outlined in the Clontech Yeast Protocols Handbook were then used to monitor growth, transformation, and β-galactosidase activities in the yeast.

### BiFC Assays

The AtHUB2 and GmHUB2 coding regions were cloned into the pVYCE(R) vector such that they were connected to the YFP C-terminus, whereas GmUBC9 was cloned into the pVYNE(R) vector such that it was fused to the YFP N-terminus (Waadt and Kudla, 2008). Recombinant vectors were then co-transformed into *Arabidopsis* protoplasts in pairs, with DAPI being utilized to monitor localization to the nucleus (Tang et al., 2016). The fluorescence signal was observed using a Carl Zeiss LSM 700 confocal microscope.

### Immunoblotting Assays

Root and leaf samples were first homogenized with liquid nitrogen, after which a digestion buffer (50 mM Tris-HCl, pH 7.5, 150 mM NaCl, 0.1% NP-40, 4 M carbamide, 1 mM PMSF) was used to extract protein from these samples. A Coomassie protein assay kit (Bradford) was then used to quantify protein levels in these samples, after which they were analyzed *via* 12% SDS-PAGE and immunoblotted with appropriate antibodies, including: Mouse anti-H2Bub1, Millipore, 05-1312; Mouse anti-H2B antibody (Abcam, ab52484); Goat anti-mouse IgG H&L (HRP) (Abcam, ab205719).

## Results

### Genome-Wide Analysis of 91 UBC-Like Members in Soybean: Identification and Distribution

**Figure 1:**
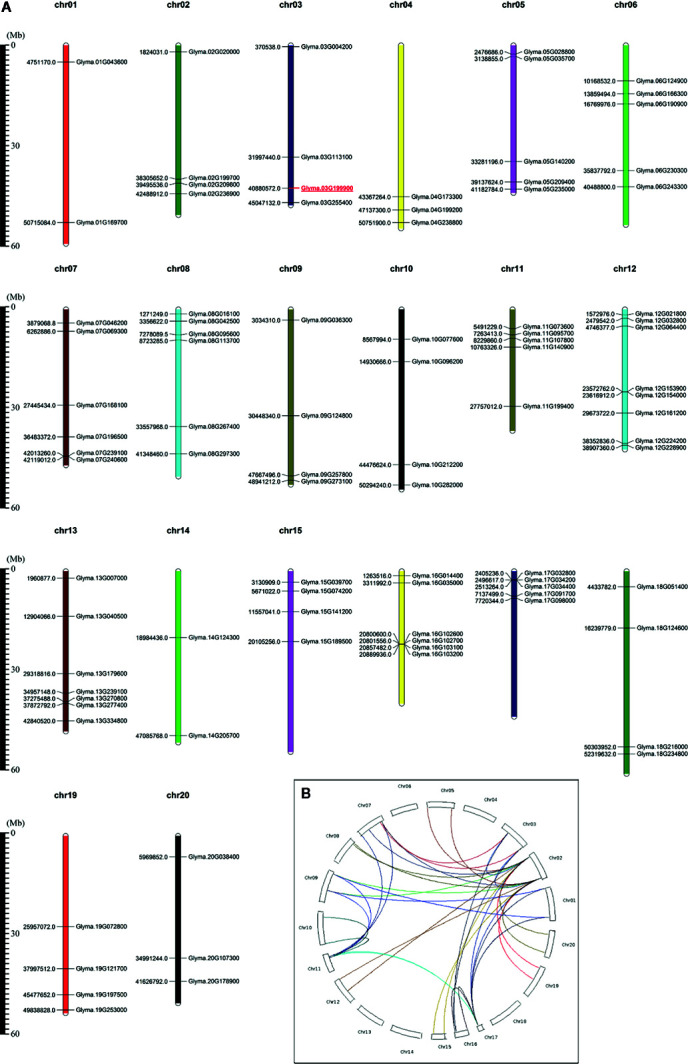
Soybean *UBC* gene locations and duplications. **(A)** Soybean *UBC* gene locations on specific chromosomes. Phytozome v10.3 was used to assess the chromosomal locations of different soybean *UBC* genes, with the scale bar being shown in megabases (Mb). Chr, chromosome. **(B)** WGD/segmental duplications of 91 soybean *UBC* genes. WGD/segmentally duplicated *GmUBC* gene pairs are linked by different colored lines. The illustration was generated using CIRCOS software.

The sequences of known UBC proteins from *Arabidopsis* were retrieved from the TAIR database. These sequences were used as queries to search against the soybean genome database using the BLASTP program with a threshold *E*-value cutoff of 1.0 × e^−5^ (Espín et al., 2012). Then, each candidate soybean UBC sequence used as a query against the Pfam database to confirm its membership in the UBC family (Finn et al., 2016). Repeat sequences were removed manually (Zhang et al., 2018; Li et al., 2019). These predicted proteins exhibited significant diversity with respect to their predicted Mw, *p*I, and sequences (**Supplementary Table S1**). We next utilized the Phytozome database (www.phytozome.net/) in order to download genomic, protein, and upstream nucleotide sequences for each of these 91 GmUBCs. The predicted protein sequences for these GmUBCs ranged between 64 amino acids (GmUBC57) and 1124 amino acids (GmUBC90) in length, with Mw values from 7.53167 to 123.66238 kDa. Predicted *p*I values ranged from 4.26 (GmUBC30) to 9.65 (GmUBC28). GmUBCs have larger ranges of seize, weight and pI than those of the ubiquitin E1 proteins in *Arabidopsis* (length (aa.) about 1,077–1,080, weight about 120KDa and *p*I about 4.9) (Hatfield et al., 1997). *Glycine max (Linn.) Merr.* has twenty chromosomes between the sizes of 34.8 Mb (chromosome 11) and 58.0 Mb (chromosome 18). We mapped the locations of these 91 *GmUBC*s onto these twenty chromosomes (**Figure 1**), with specific chromosomal locations being detailed in **Supplementary Table S1**.

Gene duplication events that occurred during the evolution of genomes are considered as the major mechanisms that contributed to the complexities of genomes and the expansion of gene families in plants (Wang et al., 2017). In order to get more information about the mechanisms that contributed to the expansion of the *GmUBC* genes, the occurrence of whole genome duplication (WGD)/segmental duplication and small-scale (local) tandem duplication events in soybean *UBC* genes were examined (**Figure 1B**, **Supplementary Table S6**). Many *GmUBC* genes (33 out of 91 genes, 36.26%) have a one-pair duplication. Interestingly, there are two *GmUBC* (2 out of 91genes, 2.20%) genes have more than one-pair of duplicates, for example, *GmUBC24* has three duplicated genes and *GmUBC70* has two duplicated genes (**Supplementary Table S6**).

### Phylogenetic Relationships and Structural Analysis of UBC-Like Proteins in Soybean

**Figure 2:**
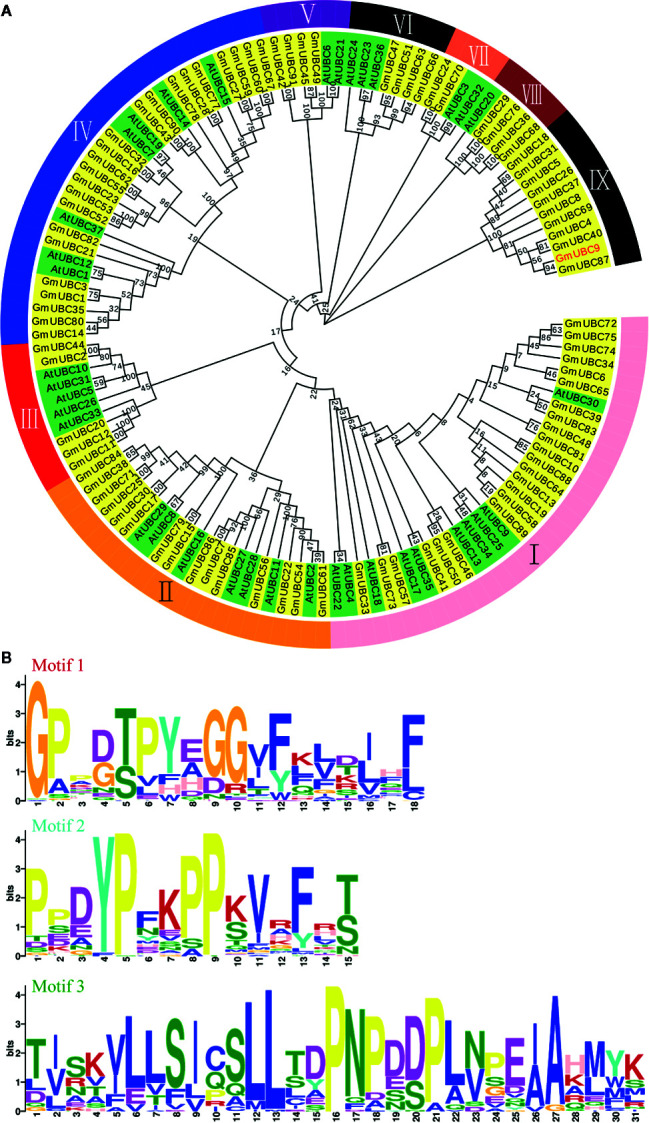
A phylogenetic tree of 110 plant UBC proteins and representative motifs of different GmUBC protein classes for the 91 soybean UBCs. **(A)** Phylogenetic analyses of UBCs from soybean and *Arabidopsis*. The nine groups corresponding to nine branches are indicated with numbers (IX. Full-length UBC amino acid sequences were aligned using Clustal X 2.0, with MEGA 6.0 being used to construct a phylogenetic tree *via* a neighbor-joining approach (1,000 boostrap replicates). **(B)** Graphical sequence logo of discovered motifs of 91 GmUBCs.

Phylogenetic analysis was used to classify the 91 GmUBC proteins and 37 total *Arabidopsis thaliana* UBC proteins into nine groups (I-IX **Figure 2A**). In an effort to compare domain-based classifications of these proteins with our sequence-based Neighbor–Joining (NJ) tree, the yellow block represents GmUBC and the green block represents AtUBC. Interestingly, in the group IX there are 11 members of soybean UBCs, but there are no members of *Arabidopsis* UBCs. This is different from the other eight groups.

Based on the alignment of the UBC domains from the above 91 proteins, a graphical sequence logo representing the sequence patterns was generated using MEME online (**Figure 2B**) and the complete alignment information was shown in **Supplementary Table S7**. In this analysis, we discovered three motifs. The E_value of Motif 1 was 1.8e-706, and the width of it was 18 aa. The E_value of Motif 2 was 1.8e-624, and the width of it was 15 aa. The E_value of Motif 3 was 3.0e-762, and the width of it was 31 aa. Most of 91 GmUBCs have these three Motifs.

**Figure 3:**
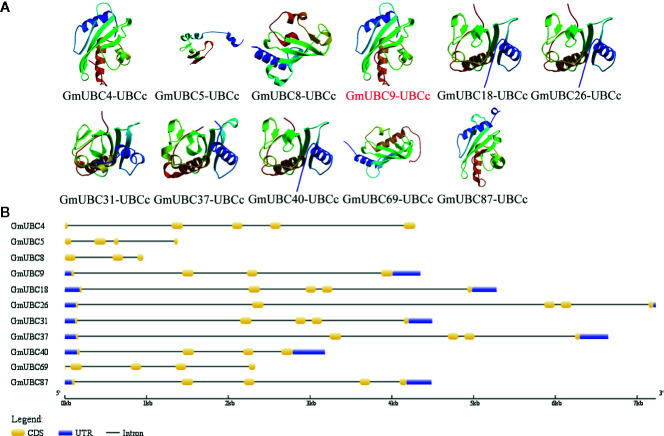
The structures of UBCc domain and exon-intron in soybean Group IX UBCs. **(A)** Predicted domain structures for soybean Class I UBCs, as analzed using SMART (http://smart.embl-heidelberg.de/) and drawn using ExPAsy (https://swissmodel.expasy.org/). **(B)** Soybean *UBC* gene intron–exon structures were assessed with the online Gene Structure Display Server tool.

All 11 group IX soybean UBC proteins have a UBCc domain (**Figure 3A**). All UBCc domains include α-helices and β-strands. In terms of spatial structure, apart from GmUBC5, GmUBC18, GmUBC26, GmUBC31, GmUBC37, and GmUBC40, GmUBC4, GmUBC9, and GmUBC87 are similar to each other in predicted 3D structure, and so are GmUBC8 and GmUBC69. The Gene Structure Display Server (http://gsds.cbi.pku.edu.cn/) was used to more fully characterize 11 selected GmUBCs, with their genomic and coding sequences (CDS) being submitted to yield basic information (**Supplementary Table S1**). The intron-exon structures of these genes were determined (**Figure 3B**). These findings revealed that phylogenetically-related *UBC* genes may have similar a gene structure. For example, *GmUBC4/8/69* and *GmUBC9/18/26/31/37/40/87* had gene structures that were similar to one another, making it likely that they share a common or closely-related ancestor.

### Expression Patterns of *UBC*-Like Genes in Soybean

**Figure 4:**
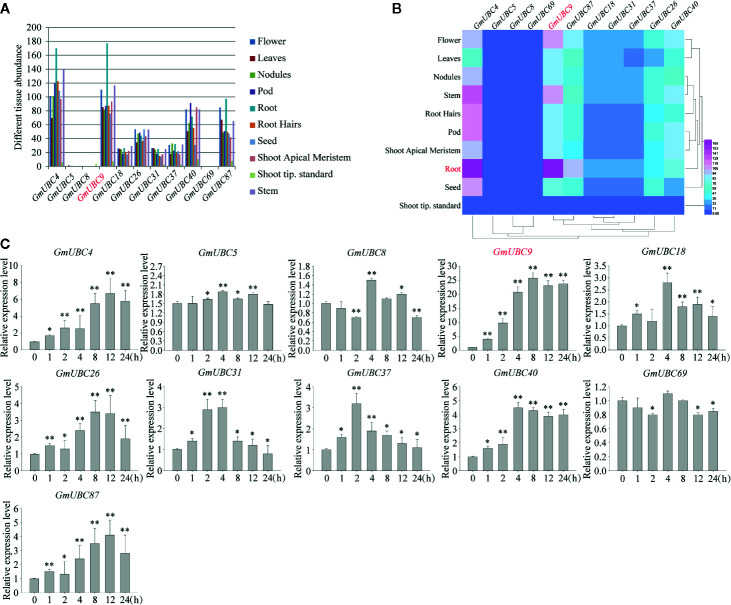
Differential expression analyses of soybean Class I *UBC* family members. **(A)** SoyBase was used to download transciptomic data pertaining to soybeans, with genes that were transcriptionally active being those with 2+ sequence reads in 1+ tissues or developmental stages. **(B)** SoyBase (https://www.soybase.org/soyseq/) data from an Affymetrix soybean gene chip assay that incorporated 10 soybean *UBCs* in the different tissues and development stages were used for analyses, with the HemI software being used generate heatmaps. **(C)** Soybean *UBC* gene expression profiles in response to drought were assessed, with *CYP2* used for normalization. Data are means and standard deviations from three biological replicates. *P < 0.05; **P < 0.01 vs. controls; Student’s t-test.

Compared with other groups, only group IX does not contain AtUBC. Interestingly, the 11 *GmUBC* genes chosen here were absent from the paper (Zhang et al., 2018) in which transcript for 50 UBC genes were detected in at least one of the 14 soybean plant tissues examined using the Soybase data. Therefore, we chose group IX as the research object. To assess the patterns of gene expression associated with these 11 group IX Gm*UBCs* across a range of tissue types, we next downloaded extant data from the soybean genome database Phytozome (quick link: PhytoMine/Expression Quantification). Data analysis of the tissues and organs revealed that *GmUBC4* and *GmUBC9* were expressed at high levels in all tissues, especially in roots, while *GmUBC5*, *GmUBC8* and *GmUBC69* were hardly expressed, and *GmUBC18*, *GmUBC31* and *GmUBC37* had a low level of expression in soybean (**Figure 4A**).

The GmUBC expression pattern heatmap was produced using HemI (**Figure 4B**). In addition to further showing the expression patterns of *GmUBCs* in different tissues, this heat map also provided a clustering analysis of these genes. The heatmap showed clustering of *GmUBC9* and *GmUBC87*, which indicated that the expression patterns of *GmUBC9* and *GmUBC87* were highly similar. In other words, we could intuitively see that *GmUBC9* and *GmUBC87* converge to the same branch in **Figure 4B**, and their expression levels are similar in different parts of soybean tissues. In addition, *GmUBC26* and *GmUBC40* also had a similar expression pattern and the expression of *GmUBC4* in the root was also obvious.

Due to a *UBC2* of soybean cultivar line WF7 confers enhanced drought tolerance in *Arabidopsis* (Zhou et al., 2010), these 11 soybean *UBC* genes were additionally chosen for further investigations of how they responded to drought conditions, with RT-qPCR being used to assess plants after a 0, 1, 2, 4, 8, 12, and 24h drought exposure. Unlike RNA-seq was done on plants 1, 6, and 12h under dehydration (Belamkar et al., 2014), this method can get more comprehensive information. These genes exhibited diverse expression patterns under drought (**Figure 4C**): 6 genes (*GmUBC4/9/26/37/40/87*) were up-regulated (>3-fold) by drought stress. Among these six genes, the expression level of *GmUBC9* had the most significant increase, reaching about 25-fold higher compared to pre-drought levels. The remaining four genes were all slightly upregulated by drought treatment. Based on this result, we chose *GmUBC9* as the target gene for subsequent research.

### GmUBC9 Localizes to the Nucleus and Endoplasmic Reticulum

We firstly predicted the nuclear localization signal (NLS) of GmUBC9 by PredictNLS (https://rostlab.org/owiki/index.php/PredictNLS) (**Supplementary Figures S7A, B**) and PSORTII (https://psort.hgc.jp/form2.html) (**Supplementary Figure S7C**). PredictNLS predicted that GmUBC9 had an NLS and functioned in the nucleus. Results of the *k-NN* prediction by PSORTII showed that there is a 47.8% predicted probability of GmUBC9 localizing in the cytoplasm and a 21.7% predicted probability of GmUBC9 localizing in the nucleus. These results suggested that GmUBC9-GFP fusion proteins localized to the nucleus and endoplasmic reticulum.

**Figure 5:**
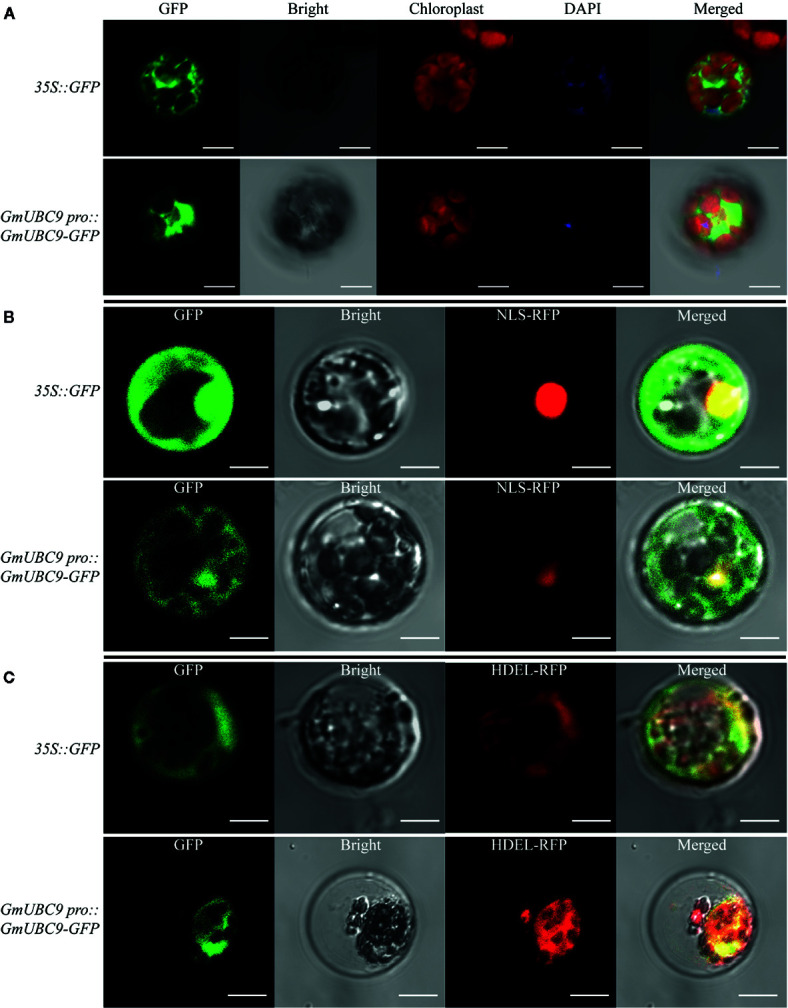
Subcellular GmUBC9 localization in *Arabidopsis* protoplast, with *35S::GFP* as a control, DAPI **(A)** together with nuclear localization signal-RFP (NLS-RFP) **(B)** was used as a nuclear marker and endoplasmic reticulum localization signal-RFP (HDEL-RFP) **(C)** was used as an ER marker. Confocal laser scanning microscopy was used to assess fluorescence. Scale bar = 10 μm.

We next evaluated the subcellular localization of *GmUBC9* using GFP-tagged protein expression vectors, such that the *GmUBC9* coding region was fused to the GFP N-terminus while remaining under endogenous promoter control. We then utilized confocal microscopy to monitor GFP localization within of *Arabidopsis* mesophyll cell protoplasts at 16 h post-transformation mediated by PEG. As a control, cells were instead transformed using *35S::GFP*. We used two nuclear markers, DAPI nuclear fluorescent dye (**Figure 5A**) and nuclear localization signal tagged with RFP (NLS-RFP; **Figure 5B**), and one endoplasmic reticulum marker (HDEL-RFP; **Figure 5C**). Control GFP exhibited uniform distribution in the mesophyll cell protoplast (**Figure 5**), while GmUBC9-GFP fusion proteins localized to the nucleus and endoplasmic reticulum.

In order to further confirm the true subcellular localization of GmUBC9 in soybean cells, we observed the localization of GmUBC9-GFP under the normal growth conditions of soybean with a laser confocal microscope (**Supplementary Figure S3**). The results showed that GmUBC9 was expressed in the root cap, meristem, elongation area and root hair of the root tip. Among them, meristem region and elongation region have the most abundant expressions. In addition, the green fluorescence of GFP overlaps with the fluorescence signal of mCherry, suggesting that GmUBC9 is on ER. This again confirms the previous results.

### *GmUBC9* Overexpression Enhances *Arabidopsis* Drought Stress Tolerance

**Figure 6:**
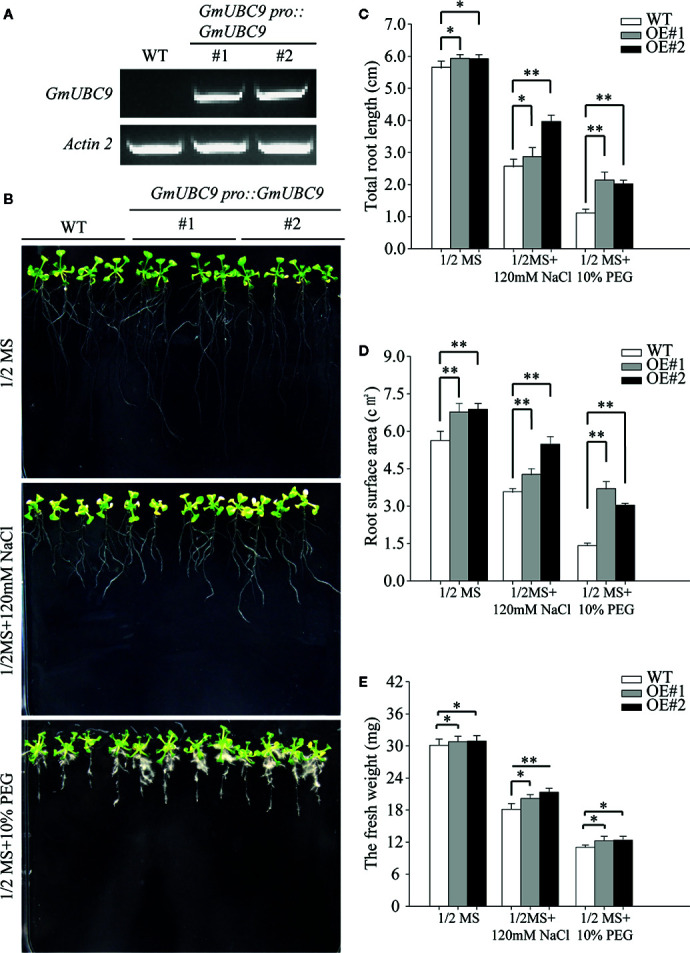
Heterologous *GmUBC9* overexpression in *Arabidopsis* improve tolerance to drought conditions. **(A)** *GmUBC9* levels in T3 transgenic *Arabidopsis* plants were assessed *via* semi-quantitative RT-PCR. **(B)** WT and transgenic *Arabidopsis* responses to treatment with PEG and NaCl. Images were collected 8 days post-treatment. **(C)** Total root length, **(D)** root surface, and **(E)** fresh weight of WT and transgenic lines. All values are presented as means of three independent biological replicates. Data are means and standard deviations. *P < 0.05; **P < 0.01 vs. controls; Student’s t-test.

Given the identification of the overexpression of *GmUBC9* increased during drought exposure, we next assessed the tolerance of transgenic *Arabidopsis* lines for salt and drought stress in medium and soil. First, we screened the obtained transgenic Arabidopsis strains. We transferred 7-day-old uniformly-germinated seeds onto 1/2 MS medium. When they grow to four leaves, total RNA samples (the whole plant) were extracted using a RNeasy Plant Mini Kit with on column DNase treatment (RNase-free DNase set, QIAGEN). cDNA was synthesized from 2 mg of total RNA using the transcription first-strand cDNA synthesis kit (Promega). Semi-quantitative RT-PCR of *GmUBC9* genes was conducted with gene-specific primers, and *Arabidopsis actin2* (*AtACT2*) (*At3g18780*) gene was used as an internal control. Results of semi-quantitative RT-PCR are shown in **Figure 6A**. Based on these findings, we selected the OE-1 and OE-2 T_3_ transgenic *Arabidopsis* lines that expressed *GmUBC9* at high levels for downstream experimentation (**Supplementary Figure S4**). When seedlings were grown in medium, PEG6000 was employed as a means of simulating drought stress. The functional role of *GmUBC9* under drought stress conditions was evaluated *via* transferring 7-day-old uniformly germinated WT and *GmUBC9*-transgenic lines to 1/2 MS medium supplemented with 10% PEG6000. Following a one-week treatment period, we observed longer roots, larger root surfaces, and greater fresh weights for transgenic plants relative to WT controls (**Figures 6C–E**). An Expression 11000XL root system scanning analyzer (Epson, Nagano, Japan) fitted with a transparency adapter and calibrated by Regent Instruments, Inc. was used to scan roots. The root system WinRHIZO 2016 program (Regent Instruments, Inc., Quebec, Canada) was used to analyze root data (Kraft and Boge, 2001). Under normal conditions, we observed no differences between WT and transgenic plants (**Figures 6B–E**). In addition, we also found that under salt treatment, root growth of the *GmUBC9*-overexpressing lines was better than that of WT (**Figure 6B**). However, the difference was smaller than that observed for drought treatment (**Figures 6C–E**). Therefore, we focused on the mechanism of drought resistance in which *GmUBC9* is involved.

**Figure 7:**
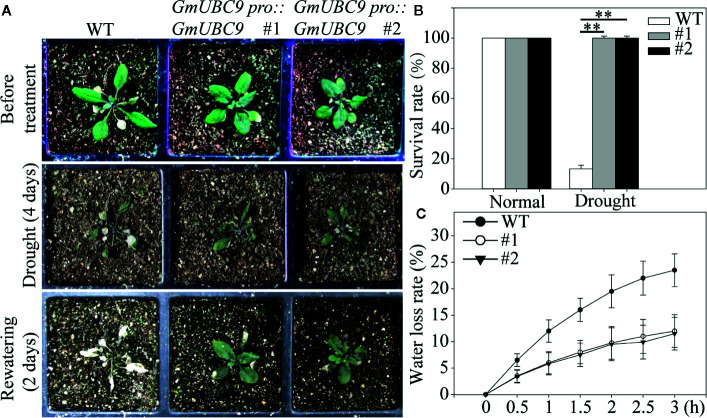
Drought tolerance phenotypes of WT and *GmUBC9* transgenic *Arabidopsis* in soil. In **(A)**, these lines were planted in a large tray separated by a small compartment. We counted survival rate **(B)** and water loss rate **(C)**. Data are presented as the mean ± SD of three independent biological replicates. Data are means and standard deviations. **P < 0.01 vs. WT; Student’s t-test.

The ability of *GmUBC9* transgenic plants to tolerate drought stress was assessed by withholding water from 3-week-old WT and *GmUBC9* transgenic *Arabidopsis* seedlings for a 4-day period. Under normal conditions, the leaves of overexpressed lines were arranged more closely than those of the wild type (**Figure 7A**). In contrast, under drought stress conditions we found that *GmUBC9*-overexpressing plants presented with a higher survival rate and a lower rate of water loss relative to WT controls (**Figures 7B, C**). The *GmUBC9*-overexpressing lines showed excellent drought resistance.

### Overexpression of GmUBC9 Improved Soybean Drought Tolerance

**Figure 8:**
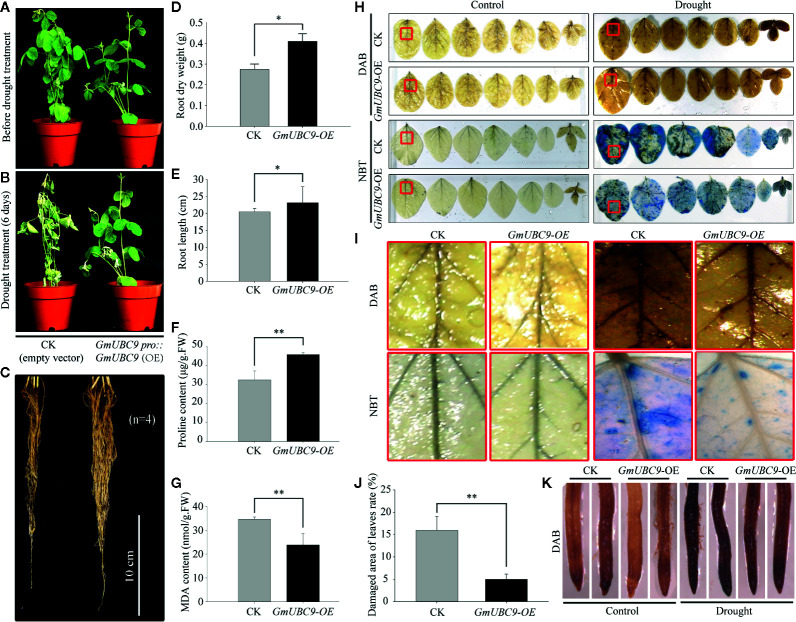
*GmUBC9* enhances soybean hairy root drought tolerance. Seedlings (2–5 cm roots) were initially grown for 5 days under normal growth conditions, after which water was withheld for 6 days. Drought resistance phenotypes in CK and OE plants under drought conditions are shown **(A)** in aerial tissues before drought, **(B)** in aerial tissues after drought, and **(C)** in roots after drought. Root dry weight **(D)** and root length **(E)** of the water-stressed plants was monitored. The leaf contents of proline **(F)** and MDA **(G)** were detected in CK and OE plants under drought. DAB and NBT **(H**, **I)** CK and OE plant leaf staining following exposure to normal or drought or conditions for 6 days. Color depth corresponds to H_2_O_2_ and O_2_^−^ levels in leaves **(H**, **I)**. The percentage of damaged area of the leaves of CK and OE plants following exposure to normal or drought or conditions for 6 days. **(J)**. DAB **(K)** CK and OE plant root staining following exposure to normal or drought or conditions for 6 days. The data were means ± SDs of four independent biological replicates (n=4). Data are means and standard deviations from. *P < 0.05; **P < 0.01 vs. controls; Student’s t-test.

As an additional means of evaluating the role of *GmUBC9* in soybean drought tolerance, we employed an *A. rhizogenes*-mediated soybean hairy root assay approach as detailed in prior studies (Kereszt et al., 2007; Hao et al., 2011). Briefly, we infected 1-week old soybean seedlings using empty pCAMBIA3301 (CK) and *GmUBC9*-OE constructs, after which hairy root samples from these plants were collected and analyzed *via* RT-qPCR, which confirmed that *GmUBC9-*OE plants exhibited much higher *GmUBC9* expression than did CK lines (**Supplementary Figure S6**). As was shown in **Supplementary Figure S6**, the expression level of the three overexpression lines was similar. Then the experiment was made with the line 1. Main roots were then removed, and water was withheld to simulate drought. We observed comparable soybean phenotypes when normal conditions were used (**Figure 8A**), whereas in response to drought stress, the CK lines showed symptoms typical of drought stress, such as wilting, leaf chlorosis, and leaf abscission, unlike the *GmUBC9*-OE lines (**Figure 8B**). We found that drought-stressed *GmUBC9*-OE roots were longer and stronger compared to the drought-stressed CK roots (**Figure 8C**). In addition, the root dry weight and root length data showed that root development in the *GmUBC9*-OE lines was better than that of CK during drought treatment (**Figures 8D, E**). Following a 6-day period during which water was withheld, we compared the levels of MDA and proline, which are important stress-associated metabolites, between transgenic and CK seedlings. We found that transgenic seedlings exhibited markedly higher level of proline and lower level of MDA in response to drought stress, respectively (**Figures 8F, G**). All these findings suggested that *GmUBC9* may enhance drought stress tolerance.

Reactive oxygen species (ROS) are induced by physiological stress and the growth and development of plants. To assess the presence and distribution of ROS, DAB and NBT were used to stain soybean leaves as a means of detecting H_2_O_2_ and O_2_^−^ contents in the context of either normal or drought stress conditions in both our control and *GmUBC9*-OE lines. Under water deficit, OE plants exhibited a reduced staining color depth relative to CK plants (**Figures 8H, I, K**). In addition, Image J (https://imagej.nih.gov/ij/) was employed for calculating the damaged area ratio of leaves of CK and OE plants (**Figure 8J**). After the image is grayed with Image J, the damaged area will be black, while the undamaged area will be white. The ratio of damaged is black as a percentage of the total area. This experiment involves three repetitions. The results showed that the ratio of damaged leaf area in CK plants was higher than that of the OE lines (15% vs 5%, respectively). In addition, DAB staining results of root tips showed that CK was darker than OE lines before and after drought treatment (**Figures 8K**). These findings suggested that H_2_O_2_ and O_2_^−^ levels in CK plants were elevated relative to OE plants, and thus overexpression of *GmUBC9* reduced the concentration of H_2_O_2_ and O_2_^−^.

### GmUBC9 Interacted With HUB2, an E3 Ubiquitin Ligase, in Soybean and *Arabidopsis*

**Figure 9:**
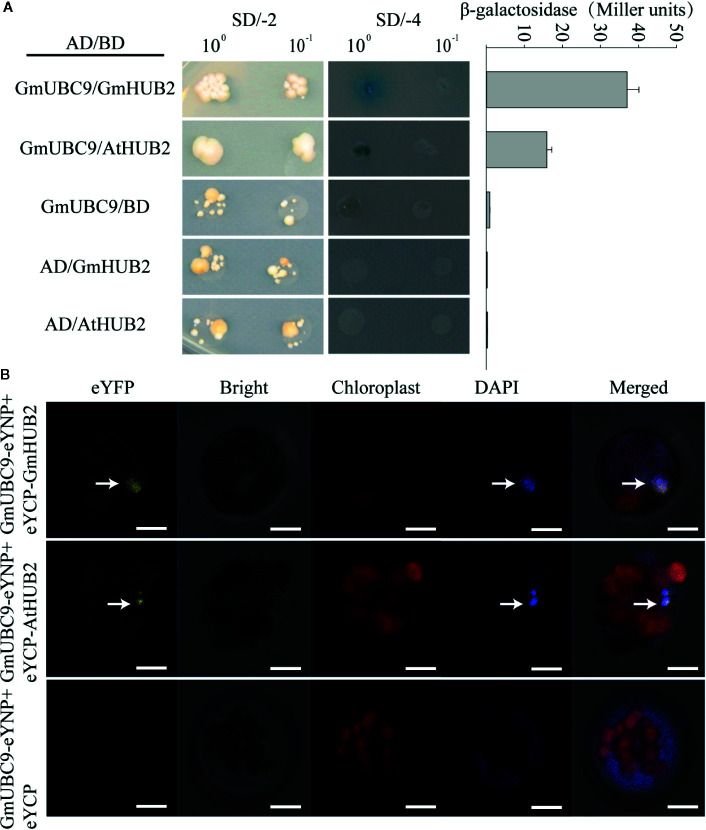
GmUBC9 interacts with GmHUB2/AtHUB2. **(A)** Interaction between GmUBC9 and GmHUB2/AtHUB2 detected by yeast two-hybrid (Y2H) assays and a quantitative assay for β-galactosidase activity. The presented values are the averages of four independent assays with standard deviations. pGADT7 (AD) and pGBKT7 (BD) were used as negative controls. AD, GAL4 activation domain. BD, GAL4 DNA-binding domain. **(B)** Confirmation of the interaction of GmUBC9 and GmHUB2/AtHUB2 by BiFC in *Arabidopsis* protoplasts. DAPI was used to stain nuclei. The white arrow indicates the nucleus. Scale bars, 10 μm.

In order to clarify how GmUBC9 is associated with the control of plant drought resistance and further study the regulatory mechanism of GmUBC9, we conducted Pearson Correlated Expression (An InterMine interface to data from Phytozome- https://phytozome.jgi.doe.gov/phytomine/buildBag.do) (https://phytozome.jgi.doe.gov/phytomine/portal.do?externalid=PAC:30518667&class=gene) and found 39 co-expressed genes, among which 5 genes (**Supplementary Figures S5A, B**) were most related to the expression of *GmUBC9* (**Supplementary Tables S3** and **S8**). Interestingly, all five proteins are likely to function in the nucleus (**Supplementary Tables S3** and **S8**). In addition, to study the interaction network between proteins, we used String (https://string-db.org/) to analyze the protein-protein interaction network (PPI) of GmUBC9 (**Supplementary Figure S5C**). The results showed that the interacting proteins of GmUBC9 were mostly UBC (E2) members. Next, we looked at the literature related to UBC (E2), we found a UBC interacted with a HUB2 protein that is reportedly an E3 ligase that mediates the monoubiquitination of H2B (H2Bub1) (Cao et al., 2008; Chen et al., 2019; Ma et al., 2019). We therefore evaluated potential physical interactions between GmUBC9 (Glyma.03G199900) and GmHUB2 (Glyma.02G267300)/AtHUB2 *via* yeast two hybrid assays (Y2H), and found that these proteins do indeed interact (**Figure 9A**). We also confirmed this *via* a bimolecular fluorescence complementation (BiFC) approach in *Arabidopsis*. GmUBC9 was found to interact with GmHUB2 and AtHUB2, while GmUBC9 (GmUBC9-eYNP) was unable to interact with eYCP (**Figure 9B**), suggesting these interactions to be specific. GmUBC9 and GmHUB2/AtHUB2 interactions were observed in the nucleus (**Figure 9B**) because the yellow fluorescence of YFP and the blue fluorescence of DAPI coincided.

### Overexpression of GmUBC9 in *Arabidopsis* and Soybean Enhanced H2Bub1 and Affected Expression of Some Drought Stress-Related Gene

**Figure 10:**
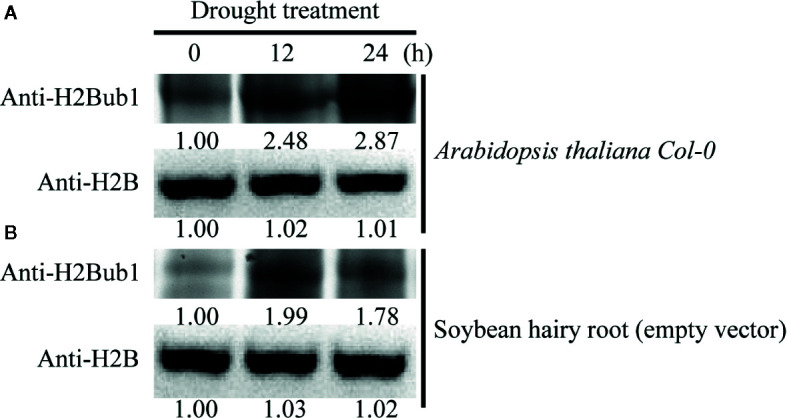
Alteration of Histone H2B monoubiquitination (H2Bub1) modification during drought response. **(A)** The H2Bub1 level detected by western blot under drought stressed *Arabidopsis* roots. **(B)** The H2Bub1 levels in soybean hairy roots were evaluated *via* western blotting. H2B served as a loading control in **(A)** and **(B)**. Band intensities normalized to the first lane to the left are shown under each band.

In light of the fact that GmUBC9 interacts with GmHUB2 and AtHUB2, which act as E3 ligases for H2Bub1 and is a key regulator of resistance to drought conditions, we next wanted to evaluate the role of H2Bub1 in general in the context of responses to drought (Cao et al., 2008; Chen et al., 2019; Ma et al., 2019). We initially determined the overall H2Bub1 levels in wild-type *Arabidopsis* Col-0 and soybean hairy root CK lines (empty vector) under severe drought conditions (~10% relative soil water content). Under these conditions, we observed significant increases in H2Bub1 levels in both *Arabidopsis* (**Figure 10A**) and soybean (**Figure 10B**).

**Figure 11:**
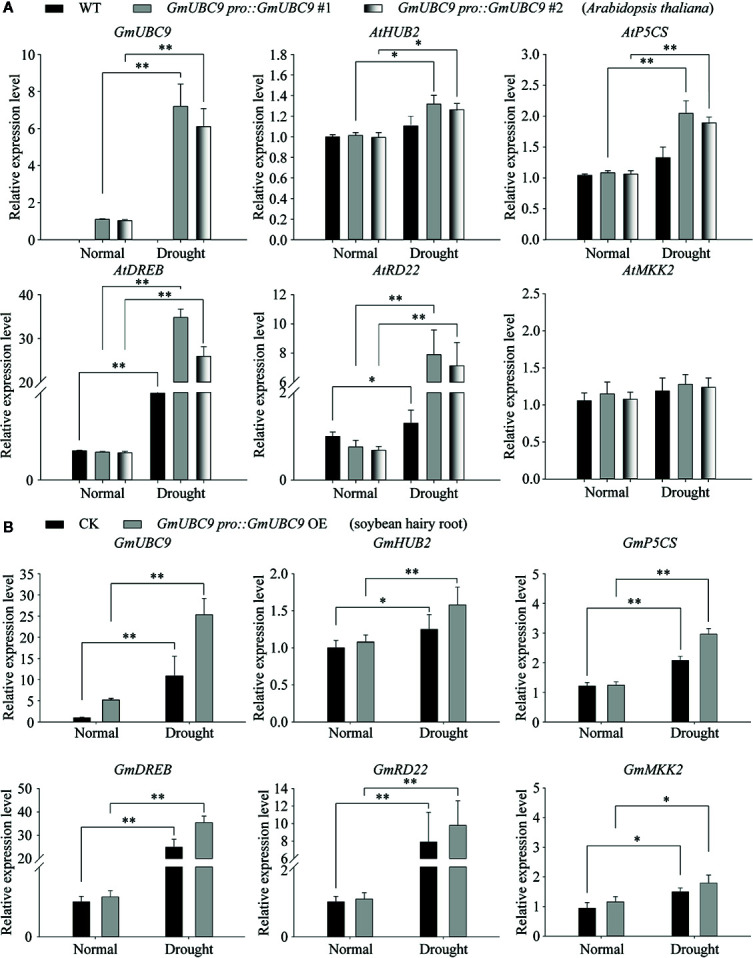
Overexpression of *GmUBC9* significantly increased the expression level of drought resistance genes in *Arabidopsis* and soybean hairy root lines under drought conditions. RT-qPCR assessment of stress-responsive gene expression in control, *GmUBC9*-overexpression *Arabidopsis* lines **(A)** and *GmUBC9*-overexpression soybean hairy root lines **(B)**. The RNA extracted from the roots of the indicated genotypes (4 weeks old) prior to or following drought treatment for 4 days. Data are normalized to *GmCYP2* expression levels. The analysis was performed with 3 independent biological replicates. Vertical bars represent the SD. (*P < 0.05; **P < 0.01; Student’s t-test).

To explore the relationship between changes in H2Bub1 levels and drought response regulation, we next assessed drought-related gene expression (Cao et al., 2008; Chen et al., 2019; Ma et al., 2019) in both *Arabidopsis* (**Figure 11A**) and soybean (**Figure 11B**). Firstly, we found that in terms of transcription, the expression level of *GmUBC9* in overexpressed soybean and Arabidopsis thaliana lines was higher than WT and the level of monoubiquitin-related gene *HUB2* in overexpressed lines was also significantly higher than WT under drought conditions (**Figures 11A, B**). The increase in their transcription level was similar to the results of the WB experiment (**Figure 10**). In the absence of drought treatment, d1-pyrroline-5-carboxylate synthetase (*P5CS*; associated with proline synthesis), dehydration-responsive element-binding protein (*DREB;* an ABA pathway-independent gene), responsive to dehydration (*RD22;* an ABA pathway-dependent gene), and mitogen-activated protein kinase 2 (*MKK2*) (Chen et al., 2019) did not exhibit differences in expression between control and transgenic plants (**Figure 11**). Following a 4 h air-drying period, we found that the expression levels of *P5CS*, *DREB*, *RD22* were increased markedly in transgenic plants than the control except *MKK2* (**Figure 11**). Together, these findings suggest that the overexpression of *GmUBC9* can markedly enhance transgenic *Arabidopsis* and soybean plant tolerance to drought conditions *via* enhancing H2Bub1 and influencing drought-related genes expression.

### Overexpression of *GmUBC9* Affected Flowering Time in Transgenic Plants

The UBC1 and UBC2 E2 conjugases in *Arabidopsis* are important regulators of plant growth and flowering (Xu et al., 2009). AtUBC1 and AtUBC2 function in concert with HUB1 and HUB2 (E3 ubiquitin ligases) in order to achieve H2B monoubiquitination. Following monoubiquitination, the possible relationship between H2B and *FLC* (the floral repressor gene *FLOWERING LOCUS C*) clade genes is observed, with H2Bub1 being essential for H3K4me3, H3K36me2, and H3K36me3 (Zhao et al., 2019) enhancement and *FLC*/*MAFs* (the floral repressor genes *MADS ASSOCIATED FLOWERING*) chromatin transcriptional activation (Cao et al., 2008). Thus, H2Bub1 plays specific roles in the regulation of the expression of important flowering gene expression in *Arabidopsis* (Cao et al., 2008).

The results of sequence analysis showed that the amino acid sequence identity of GmUBC9, AtUBC1, and AtUBC2 was as high as 65.67% (**Supplementary Figure S7D**). Domain analysis revealed that they all have a conserved UBCc domain (**Supplementary Figure S7E**). Through modeling, the spatial structures of GmUBC9, AtUBC1, and AtUBC2 was also similar (**Supplementary Figure S7F**). Therefore, we speculate that GmUBC9, AtUBC1, and AtUBC2 may also be similar in function, that is, they may share an involvement in the regulation of growth and flowering time of plants by the monoubiquitination of histones and activating the expression of flowering suppressor genes.

**Figure 12:**
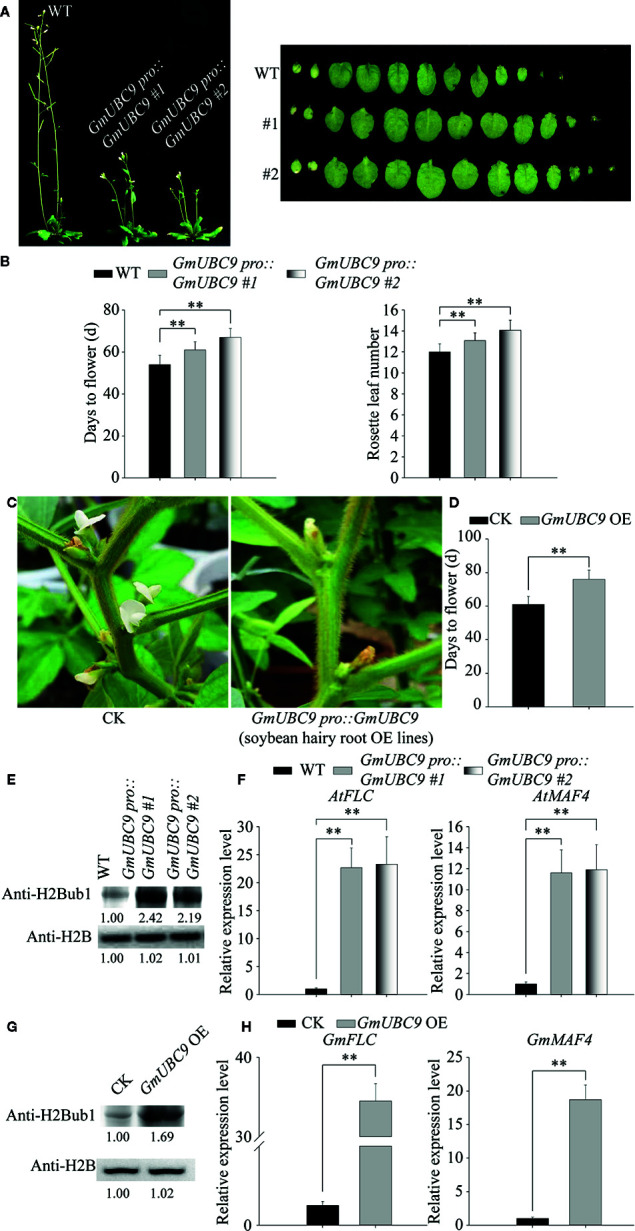
GmUBC9 inhibits plant flowering due to H2Bub1 and promotes the expression of *FLC* clade genes in *Arabidopsis*. **(A)** Flowering phenotypes and rosette leaves number of WT and *GmUBC9*-overexpressed *Arabidopsis* lines under normal growth conditions. **(B)** The number of rosettes and the number of days from germination until flowering. **(C)** Flowering phenotypes of WT and *GmUBC9*-overexpressing soybean hairy root lines under normal growth conditions. **(D)** The number of days it takes to bloom are counted. **(E)** Assessment of H2Bub1 in WT and *GmUBC9*-overexpressed *Arabidopsis* lines. The upper band was monoubiquitinated H2B, and the lower band was H2B. Values of band intensity relative to the first lanes from the left are indicated below the bands. **(F)** RT-qPCR analyses of the transcript levels of the floral repressor genes *FLOWERING LOCUS C* (*FLC*) and *MADS ASSOCIATED FLOWERING 4* (*MAF4*). Data are normalized to WT, with *AtACTIN* as an internal control. Three biological replications were performed for each experiment. Data are means with standard deviations. (**P < 0.01; Student’s t-test). **(G)** The H2Bub1 level detected by western blot in WT and GmUBC9-overexpressed soybean hairy root lines. The H2B levels served as a loading control, with normalized band intensity values being shown. **(H)** RT-qPCR analyses of the transcript levels of the floral repressor genes *FLC* and *MAF4* in soybean. Data are normalized to WT, with *AtACTIN* as an internal control. Three biological replications were performed for each experiment. Data are means with standard deviations from three independent biological replicates. (**P < 0.01; Student’s t-test).

In this study, we investigated the flowering time of the WT and *GmUBC9*-OE lines in *Arabidopsis* and found that overexpression of *GmUBC9* significantly inhibited plant growth and delayed flowering (**Figure 12A**). Wild-type and transgenic Arabidopsis plants of the same Columbia ecotype were grown at 22°C under long-day (16 h light/8 h dark). Statistics showed that the number of rosette leaves of *GmUBC9*-OE lines was more than that of WT (**Figures 12A, B**), and it took longer for *GmUBC9*-OE lines to grow from germination to flowering (**Figure 12B**). In order to further confirm the function of GmUBC9 in inhibiting flowering, we observed flowering patterns in soybean hairy root *GmUBC9*-OE lines and CK (control lines) under the same growth conditions. As shown in **Figure 12C**, the flowering of *GmUBC9* overexpression lines occurred significantly later than that of CK. CK bloomed more than a week earlier than soybean hairy root *GmUBC9*-OE lines (**Figure 12D**). Synthesizing these results, it can be concluded that the overexpression of *GmUBC9* has flowering inhibition function.

To further validate our hypothesis in terms of a molecular mechanism, we verified the global level of H2B monoubiquitination and the expression level of flowering suppressor genes in *Arabidopsis* and soybean, respectively. The results showed that after overexpression of *GmUBC9*, the levels of histone monoubiquitination in *Arabidopsis* and soybean were significantly increased, which were >2 times and >1.5 times higher than those of their respective control lines, respectively (**Figures 12E, G**). RT-PCR results showed that after overexpression of *GmUBC9*, the expression levels of flowering suppressor genes in *Arabidopsis* and soybean were significantly increased. In *Arabidopsis*, the expression levels of *AtFLC* and *AtMAF4* in overexpression lines were >20 and >10 times higher than that of control lines, respectively (**Figure 12F**). In soybean, the expression levels of *GmFLC* and *GmMAF4* in overexpression lines were >30 and >15 times higher than that of CK, respectively (**Figure 12H**).

In line with these findings, we found that *FLC* and *MAF4* transcript levels were increased in *GmUBC9*-OE lines. The *atubc1/atubc2*double loss-of-function mutants have also been shown to exhibit early flowering and reduced *MAF4, MAF5*, and *FLC* expression, in line with the roles of these genes as regulators of H2B ubiquitylation (Xu et al., 2009). Levels of H2B monoubiquitination are markedly reduced in *ubc1/ubc2* double mutants (Xu et al., 2009). Together, these results suggest that E2 GmUBC9 mediates H2B ubiquitylation, which in turn plays essential roles in governing floral repressor gene activation and other processes that result in evident *Arabidopsis* and soybean phenotypes.

## Discussion

*UBC* gene family members have been documented and characterized in a range of plants (Cao et al., 2008; Xu et al., 2009; Chen et al., 2019; Ma et al., 2019). Herein, we conducted a genome-wide assessment of *UBC* genes in soybeans, identifying 91 such putative *UBC* genes in total (**Supplementary Table S1**). E2 ubiquitin-conjugating enzyme classification is similar to that of E3 ligase gene families, and is based not on homology but on the UBCc domain and additional domains (Azevedo et al., 2001; Wang et al., 2016). Thus, the 91 GmUBC proteins were classified into five classes according to different domains/motifs as well as the UBCc domain, including a low complexity region, UBA, and a transmembrane region (**Figure 2B**). But unlike E3 ligase gene families (Wang et al., 2016), most GmUBCs have a similar biological function, mainly conferred by the UBCc motif, which is required for many fundamental processes of cell viability. Proteins destined for proteasome-mediated degradation may be ubiquitinated. Ubiquitination follows conjugation of ubiquitin to a conserved cysteine residue of UBC homologues (Yee and Goring, 2009).

We also employed a phylogenetic approach to cluster these 91 GmUBCs into five branches (**Figure 2A**), suggesting that these proteins exhibit limited sequence similarity despite having conserved motifs, potentially as a result of independent mutation accumulation over the course of evolution. Phylogenetic analyses also revealed that ten GmUBCs (**Supplementary Table S2**) exhibited a high degree of similarity to other UBCs from *Arabidopsis*. We found that GmUBC9 (Glyma.03G199900) showed the highest identity when compared to AtUBC1 and AtUBC2 (65.67%) (**Supplementary Figure S7**), which are key regulators of abiotic stress responses and flowering time (Xu et al., 2009; Chen et al., 2019). Furthermore, we found that *GmUBC9* was induced by drought stress, suggesting that *GmUBC9* may play a role in regulating plant abiotic stress responses. Interestingly, preliminary studies have been conducted on two UBC proteins in legumes (Zhou et al., 2010; Chung et al., 2013). The sequence homology alignment was shown (37.05%) in **Supplementary Figure S1**. They confirmed that the two UBCs could improve the drought resistance of plants, but they did not specify the specific molecular mechanism of UBC (Zhou et al., 2010; Chung et al., 2013).

GmUBC9 showed the highest percent identities with AtUBC1/2 (**Figure 2A**; **Supplementary Figure S6**). AtUBC1/2 is an important enzyme that mediates histone H2B monoubiquitination, thereby broadly impacting plant growth and development, such as drought stress responses and flowering (Cao et al., 2008; Xu et al., 2009; Chen et al., 2019; Ma et al., 2019). Our study confirmed that GmUBC9 has the same function in soybeans, and they are all involved in H2B monoubiquitination. This is the first report indicating that GmUBC9 regulates drought stress and flowering time in soybean by H2B monoubiquitination. We also obtained *GmUBC9*-overexpression *Arabidopsis* and soybean that demonstrated improved drought tolerance by increased plant root length under drought conditions. H2Bub1 levels can be used to assess chromatic activity in plants, and it thus play important roles in regulating transcriptional activity (Chen et al., 2019). We found that we were able to observe marked upregulation of the TFs *RD22* and *DREB*, the proline synthesis-related gene *P5CS*, and the stress-related protein kinase *MKK2* in transgenic *Arabidopsis* and soybean, while these genes had lower expression in cultivar W82 (**Figure 11**). The immunoblotting analyses revealed that the levels of H2Bub1 would be increased under drought stress (**Figure 10**). These functions are the same as those of AtUBC1/2 (Cao et al., 2008; Chen et al., 2019). Drought stress responses are complicated and entail diverse signaling and regulatory pathways. H2Bub1 may regulate gene expression indirectly or upstream, with rapid *GmDREB* responses to drought stress potentially serving as a source of signaling for additional pathways regulating gene expression.

Recent studies also have suggested AtUBC1 and AtUBC2 play important roles in plant growth and flowering control. The *Arabidopsis* homologs of *BRE1*, *HUB1*, and *HUB2*, also control H2B ubiquitylation (Fleury et al., 2007; Liu et al., 2007). In this study, we found that GmUBC9 interacts with GmHUB2 and AtHUB2 and the flowering process of *GmUBC9* overexpression lines was significantly delayed compared to the normal flowering of WT. Under normal growth conditions, the level of H2Bub1 in overexpression lines was also significantly higher than that of WT. The expression of the flowering suppressor genes *FLC* and *MAF4* in *GmUBC9*-OE lines was also significantly up-regulated (**Figure 12**). These results all confirmed that the role of GmUBC9 in regulating flowering was similar to AtUBC1 and AtUBC2 (Cao et al., 2008; Chen et al., 2019; Ma et al., 2019).

Based on the above results and previous studies, we proposed a molecular mechanism of GmUBC9 in soybean (**Supplementary Figure S8**). In *Arabidopsis* and soybean, GmUBC9 interacted with AtHUB2/GmHUB2 to monoubiquitinate H2B and regulate the expression level of downstream genes related to drought resistance and flowering. These results suggested functional diversification of UBC members in the same clade. Future analyses of will be necessary in order to assess the specific functionality of GmUBC family members *via* additional experimentation.

## Conclusion

By surveying the soybean genome, we identified 91 soybean *UBC* genes that were found to play important roles in regulating drought responses and flowering through expression analysis. GmUBC9 improved transgenic *Arabidopsis* and soybean hairy root resistance to drought conditions. Additionally, the overexpression of *GmUBC9* also inhibited the flowering of plants. Together, these findings suggest that soybean *UBC* genes may be key mediators of abiotic stress tolerance and developmental regulation in plants.

## Data Availability Statement

All datasets presented in this study are included in the article/**Supplementary Material**.

## Author Contributions

MC and H-YL coordinated the project, conceived and designed the experiments, and edited the manuscript. KC performed the experiments and wrote the first draft. W-ST, Y-BZ, Z-SX, JC, and Y-ZM contributed to valuable discussions. All authors contributed to the article and approved the submitted version.

## Funding

This work was funded by the National Key R & D Plan of China (2016YFD0101005) and National Transgenic Key Project of the Ministry of Agriculture of China (2016ZX08002002).

## Conflict of Interest

The authors declare that the research was conducted in the absence of any commercial or financial relationships that could be construed as a potential conflict of interest.

## Supplementary Material

Figure S1 An alignment of amino acid sequence and protein domains between GmUBC9 with two UBCs of previously published articles (Zhou et al., 2010; Chung et al., 2013) in legumes (soybean cultivar WF7 and *Vigna radiata*, respectively). **(A)** Alignment of the amino acid sequences of GmUBC9 and the two proteins from these articles, including “GmUBC2” and “VrUBC1”. Identical residues are shown on a black background. Blue and red boxes point to the conserved domains of the UBCc domains, respectively. **(B)** Classification of the three proteins based on their domain features. The UBCc representative protein structure for each was shown. Bar represents 100 amino acids.

Figure S2 The homology analysis of the homologous genes of *GmUBC9* in all plants. *GmUBC9* (GLYMA_03G199900) was marked in red. This analysis was done using UniProt (https://www.uniprot.org/).

Figure S3 Subcellular GmUBC9 localization in soybean hairy root. The root tip is at the top. The following four channels are root cap, meristematic zone, elongation zone and root hair respectively. The green fluorescence is GmUBC9-GFP fusion protein. The red fluorescence is ER marker protein with red mCherry protein. Confocal laser scanning microscopy was used to assess fluorescence. Scale bar = 20 µm.

Figure S4 Detection of *GmUBC9* in WT and transgenic Arabidopsis. **(A)** The GmUBC9 expression level was assessed via semi-quantitative RT-PCR in transgenic and WT plants. **(B)** Relative transcript abundance of *GmUBC9* in WT and transgenic *Arabidopsis* lines determined by RT-qPCR. The analysis was performed with 3 independent technical replicates of each transformed lines. Error bars indicate the SD of three biological replicates.

Figure S5 The prediction about proteins associated with the expression of GmUBC9 and proteins that interact with GmUBC9. **(A)** Pearson correlated expression analysis of *GmUBC9*. The correlation coefficient matrix (a part of it) of correlated genes. The red arrow points to five genes that had the highest correlation of expression with *GmUBC9* (gene ID: Glyma.20G054900, correlation coefficient = 0.90512; gene ID: Glyma.09G250300, correlation coefficient = 0.90185; gene ID: Glyma.01G154400, correlation coefficient = 0.89856; gene ID: Glyma.13G306700, correlation coefficient = 0.89856; gene ID: Glyma.07G119100, correlation coefficient = 0.88707). **(B)** Analysis of the expression differences of all relevant genes (A) in different soybean tissues and organs. The red arrow points to five genes. The data was download from Phytozome V10.3 (http://phytozome.jgi.doe.gov). **(C)** Functional interaction analysis of GmUBC9. The red arrow points to GmUBC9. We searched on the String website by protein sequence (https://string-db.org/cgi/input.pl?sessionId=ITBazYnuHwE0&input_page_active_form=single_sequence).

Figure S6 Detection of *GmUBC9* in CK (empty vector) and transgenic soybean hairy root lines. **(A)** Electrophoresis results of amplification products of vector sequences (CK lines) and target gene fragments (OE lines). **(B)** Relative transcript levels of *GmUBC9* in CK and transgenic soybean hairy root lines determined by RT-qPCR. The analysis was performed with 3 independent technical replicates of each transformed root lines. Error bars indicate the SD of three replicates.

Figure S7 Prediction of GmUBC9 signal peptides and nuclear localization signals (NLS). **(A)** The red curve represents the probability of a signal peptide (SP-Sec/SPI). Signalp-5.0 software was used for the prediction (http://www.cbs.dtu.dk/services/SignalP/). (**B–C**) The NLS prediction of GmOXR17. **(B)** This image was produced using UniProt (https://www.uniprot.org/). The red arrow points to the nucleus. **(C)** The subcellular localization prediction by PSORT II (http://www.genscript.com/tools/psort). We used the k-NN prediction. The values represent the distribution ratio of GmUBC9 in each subcellular compartment or organelle. **(D–F)** Homology analysis of GmUBC9 and AtUBC1/2. **(D)** Alignment of the amino acid sequences of GmUBC9 and AtUBC1/2. Identical residues are shown on a black background. Red box points to the conserved domains of the UBCc domains. **(E)** Classification of the three proteins based on their domain features. The UBCc representative protein structure for each one was shown. The bar represents 100 amino acids. **(F)** The predicted domain structures. The predicted structures of the UBCs were analyzed by SMART (http://smart.embl-heidelberg.de/) and were drawn online (https://swissmodel.expasy.org/).

Figure S8 Working model for GmUBC9 in the regulation of stress-responsive and flowering genes in *Arabidopsis* **(A)** and soybean **(B)**. H2Bub1 mediated by the GmUBC9 and AtHUB2/GmHUB2 complex plays pivotal roles in stress responses and regulation of flowering. GmUBC9 can interact with AtHUB2/GmHUB2 when plants are exposed to drought stress conditions and activate stress responsive genes by increasing H2Bub1 levels (**Figure 10**). In addition, the overexpression of *GmUBC9* will also increase the level of H2Bub1 and deactivate flowering (**Figure 12A, C, E & G**). An increase in H2Bub1 level will further activate the expression of downstream stress response genes and *FLC* family genes (**Figure 11**; **Figure 12F, H**) (Cao et al., 2008; Chen et al., 2019).

Figure S9 All original images of gel and Western blot.

Table S1 Detailed information of the ubiquitin-conjugating enzyme (*UBC*) genes identified in the soybean genome.

Table S2 Primary cis-acting elements in the soybean Class I *UBC* gene promoter regions. ABRE: ABA-responsive element; ARE: anaerobic-responsive element; DRE: drought-responsive element; LTR: low-temperature responsive element; MYB: response to drought and ABA signals; MYC: response to drought, ABA and cold signals; TC-rich repeat: defense and stress responsiveness.

Table S3 Pearson correlated expression analysis of *GmUBC9*. The annotation of correlated genes (an important part of it) (Supporting FIGURE S4) has been shown. Protein sequence and functional comments came from Phytozome site (https://phytozome.jgi.doe.gov/pz/portal.html#). Subcellular localization prediction was performed using PSORT II Prediction (https://psort.hgc.jp/form2.html) and Predict Protein (https://www.predictprotein.org/home).

Table S4 Primers used in this study.

Table S5 The length of the 20 chromosomes of soybean (cultivar Williams 82).

Table S6 Estimated time of the WGD/segmental duplication events among soybean *GmUBC* genes.

Table S7 Graphical sequence logo of 91 GmUBCs.

Table S8 The total 39 genes related to *GmUBC9* in Pearson Correlated Expression analysis.

## Abbreviations

UBC, ubiquitin-conjugating enzyme E2; HUB, HISTONE MONOUBIQUITINATION protein E3; H2Bub1, H2B mono-ubiquitylation; GFP, green fluorescent protein; DAPI, 4’,6-diamidino-2-phenylindole; WT, wild type; CK, the control lines; qRT-PCR, quantitative real-time PCR; Y2H, yeast two hybrid assays; BiFC, bimolecular fluorescence complementation.
