# Supplementary figures and images for: Overexpression of GmUBC9 Gene Enhances Plant Drought Resistance and Affects Flowering Time via Histone H2B Monoubiquitination
Source: Front Plant Sci. 2020 Sep 4;11:555794. doi: 10.3389/fpls.2020.555794 (PMC7498670; doi:10.3389/fpls.2020.555794)

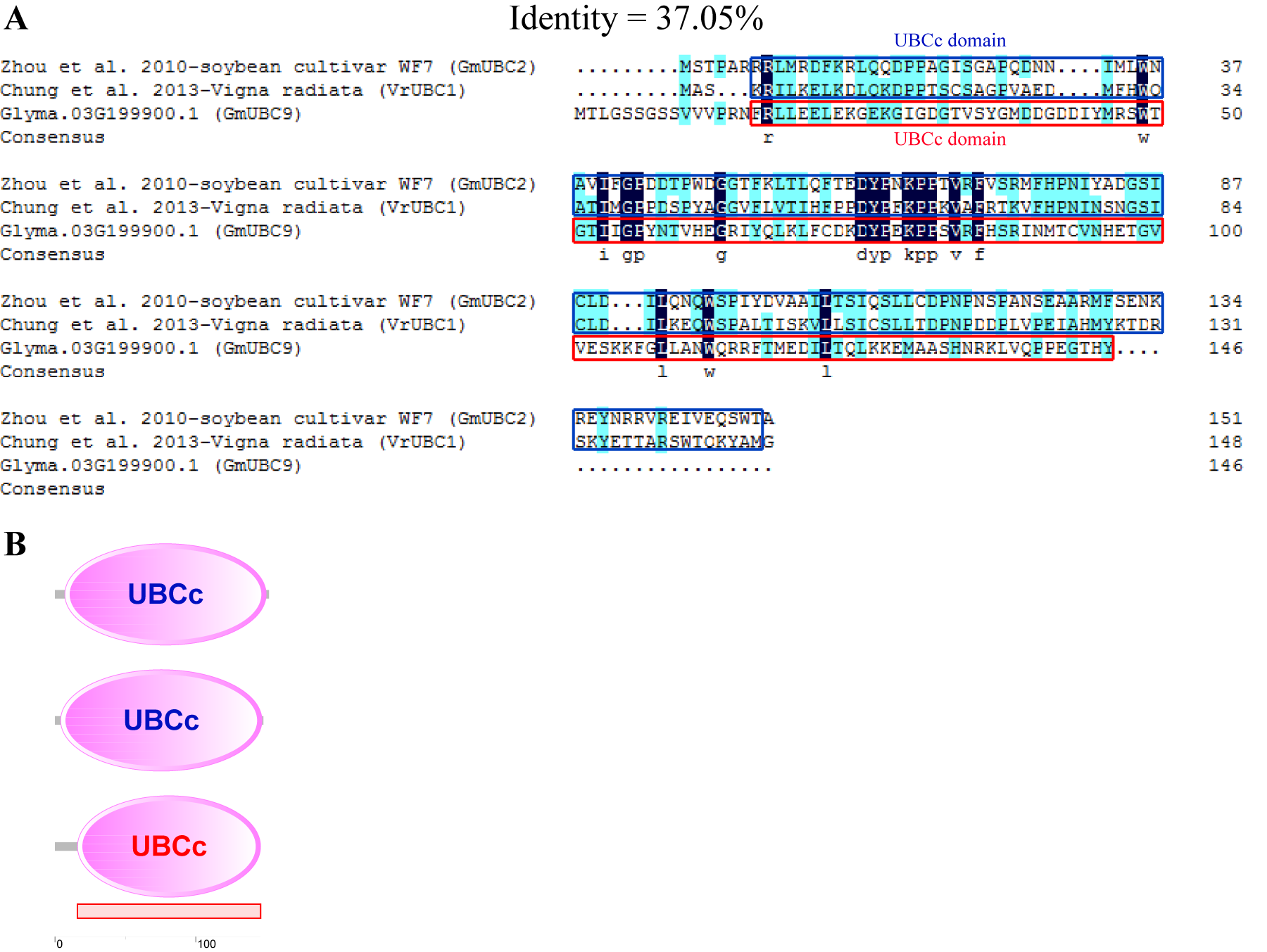

Supplement: Figure S1 — An alignment of amino acid sequence and protein domains between GmUBC9 with two UBCs of previously published articles (Zhou et al., 2010; Chung et al., 2013) in legumes (soybean cultivar WF7 and Vigna radiata, respectively). (A) Alignment of the amino acid sequences of GmUBC9 and the two proteins from these articles, including “GmUBC2” and “VrUBC1”. Identical residues are shown on a black background. Blue and red boxes point to the conserved domains of the UBCc domains, respectively. (B) Classification of the three proteins based on their domain features. The UBCc representative protein structure for each was shown. Bar represents 100 amino acids. [file Image_1.tif]

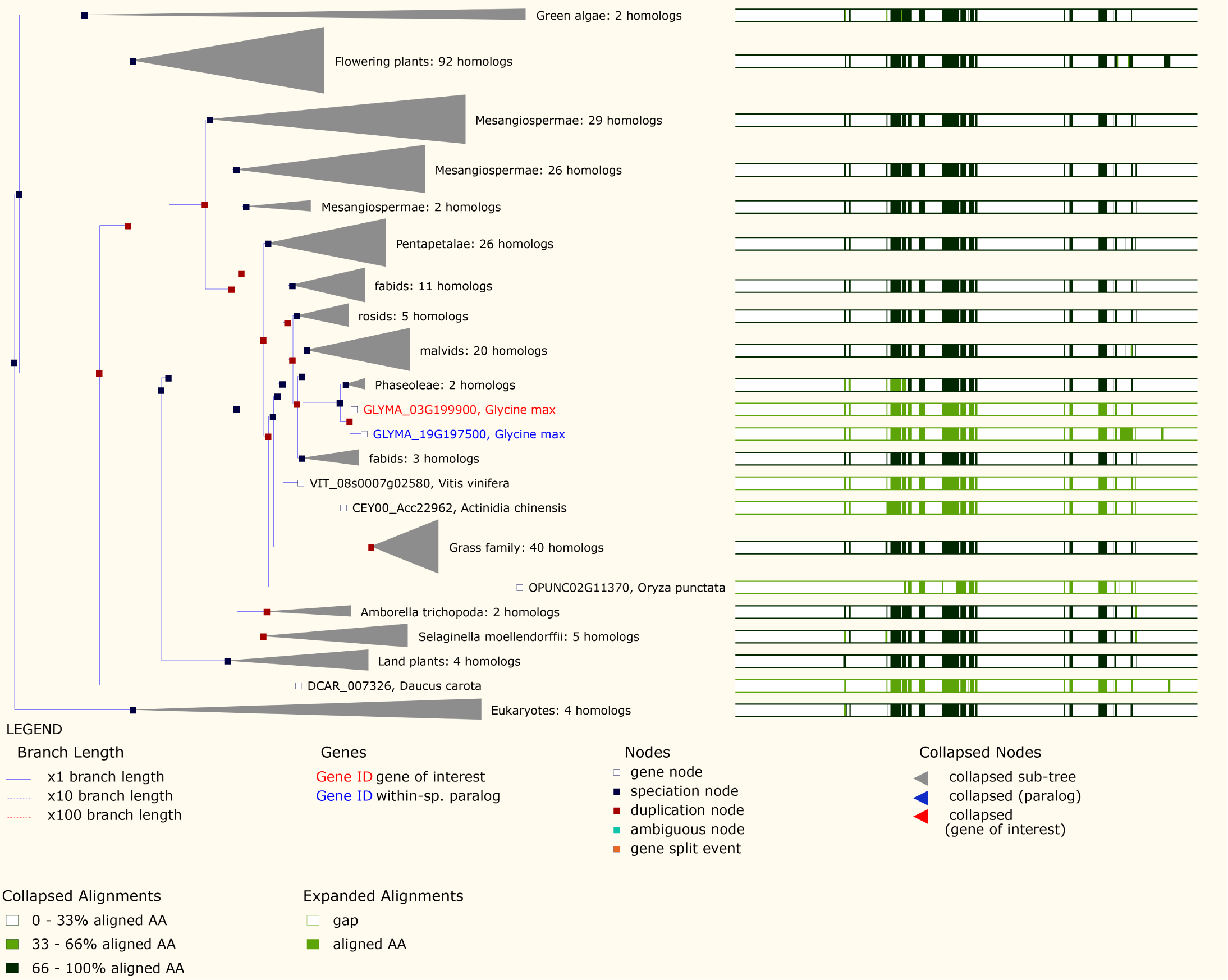

Supplement: Figure S2 — The homology analysis of the homologous genes of GmUBC9 in all plants. GmUBC9 (GLYMA_03G199900) was marked in red. This analysis was done using UniProt (https://www.uniprot.org/). [file Image_2.tif]

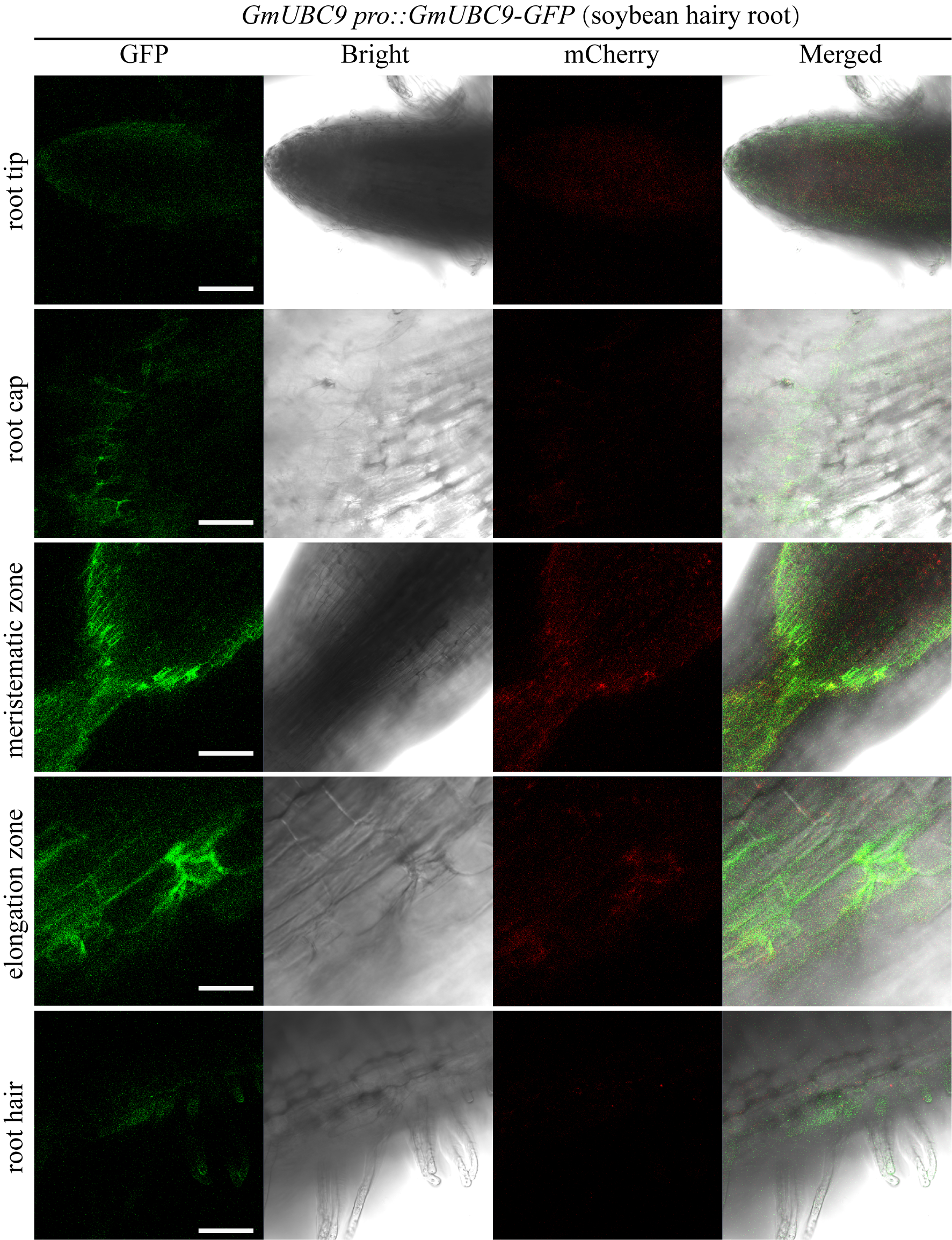

Supplement: Figure S3 — Subcellular GmUBC9 localization in soybean hairy root. The root tip is at the top. The following four channels are root cap, meristematic zone, elongation zone and root hair respectively. The green fluorescence is GmUBC9-GFP fusion protein. The red fluorescence is ER marker protein with red mCherry protein. Confocal laser scanning microscopy was used to assess fluorescence. Scale bar = 20 µm. [file Image_3.tif]

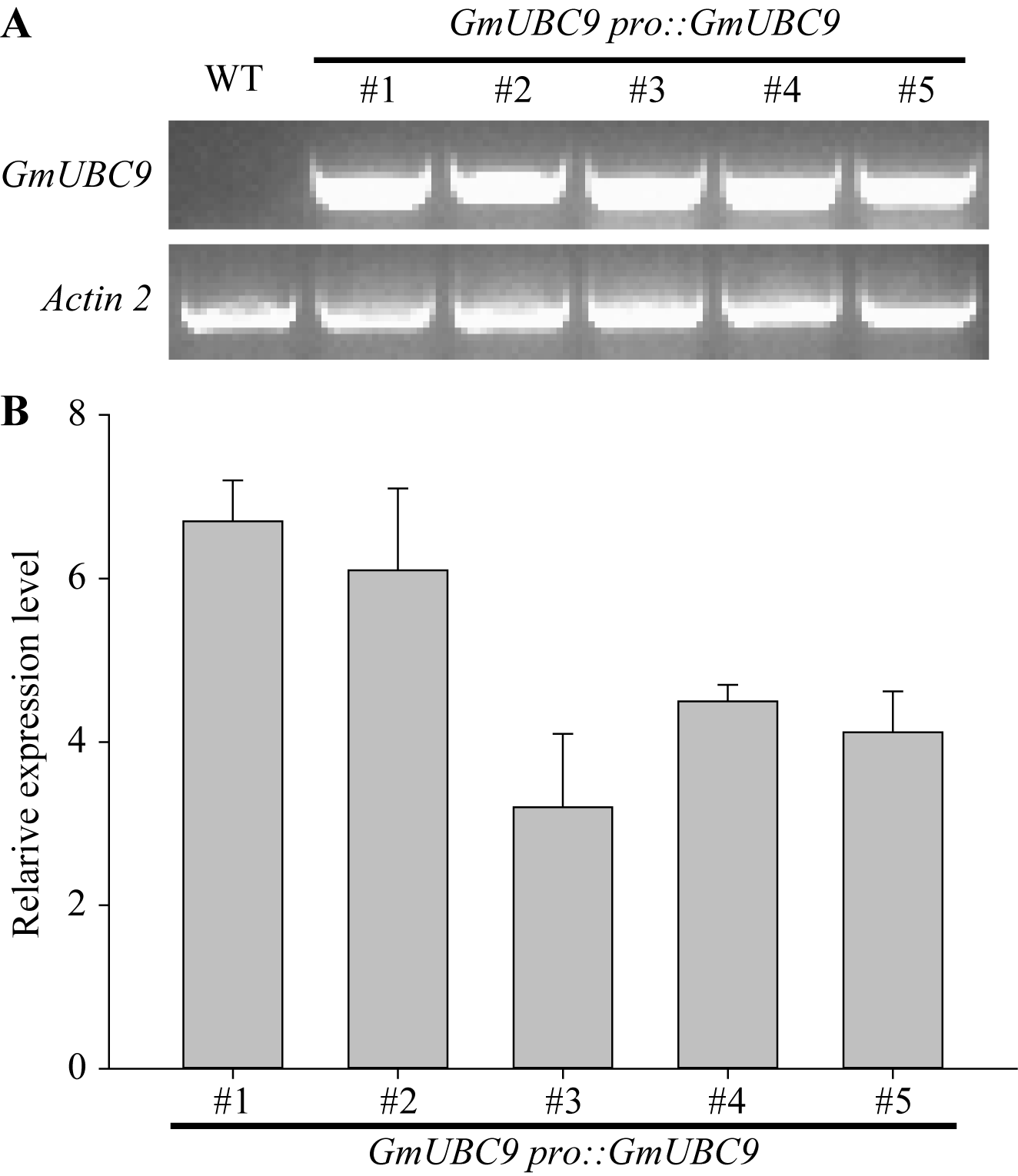

Supplement: Figure S4 — Detection of GmUBC9 in WT and transgenic Arabidopsis. (A) The GmUBC9 expression level was assessed via semi-quantitative RT-PCR in transgenic and WT plants. (B) Relative transcript abundance of GmUBC9 in WT and transgenic Arabidopsis lines determined by RT-qPCR. The analysis was performed with 3 independent technical replicates of each transformed lines. Error bars indicate the SD of three biological replicates. [file Image_4.tif]

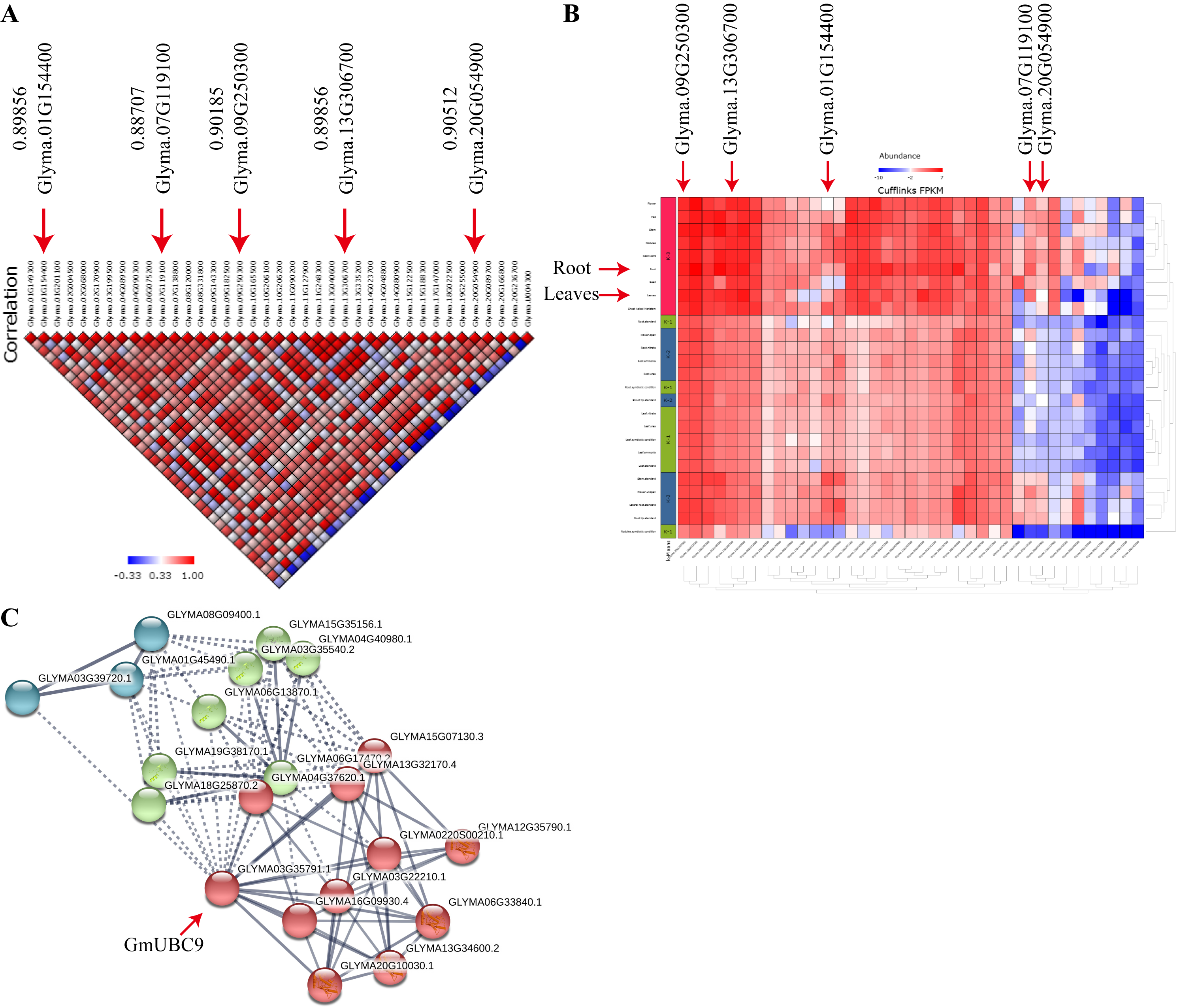

Supplement: Figure S5 — The prediction about proteins associated with the expression of GmUBC9 and proteins that interact with GmUBC9. (A) Pearson correlated expression analysis of GmUBC9. The correlation coefficient matrix (a part of it) of correlated genes. The red arrow points to five genes that had the highest correlation of expression with GmUBC9 (gene ID: Glyma.20G054900, correlation coefficient = 0.90512; gene ID: Glyma.09G250300, correlation coefficient = 0.90185; gene ID: Glyma.01G154400, correlation coefficient = 0.89856; gene ID: Glyma.13G306700, correlation coefficient = 0.89856; gene ID: Glyma.07G119100, correlation coefficient = 0.88707). (B) Analysis of the expression differences of all relevant genes (A) in different soybean tissues and organs. The red arrow points to five genes. The data was download from Phytozome V10.3 (http://phytozome.jgi.doe.gov). (C) Functional interaction analysis of GmUBC9. The red arrow points to GmUBC9. We searched on the String website by protein sequence (https://string-db.org/cgi/input.pl?sessionId=ITBazYnuHwE0&input_page_active_form=single_sequence). [file Image_5.tif]

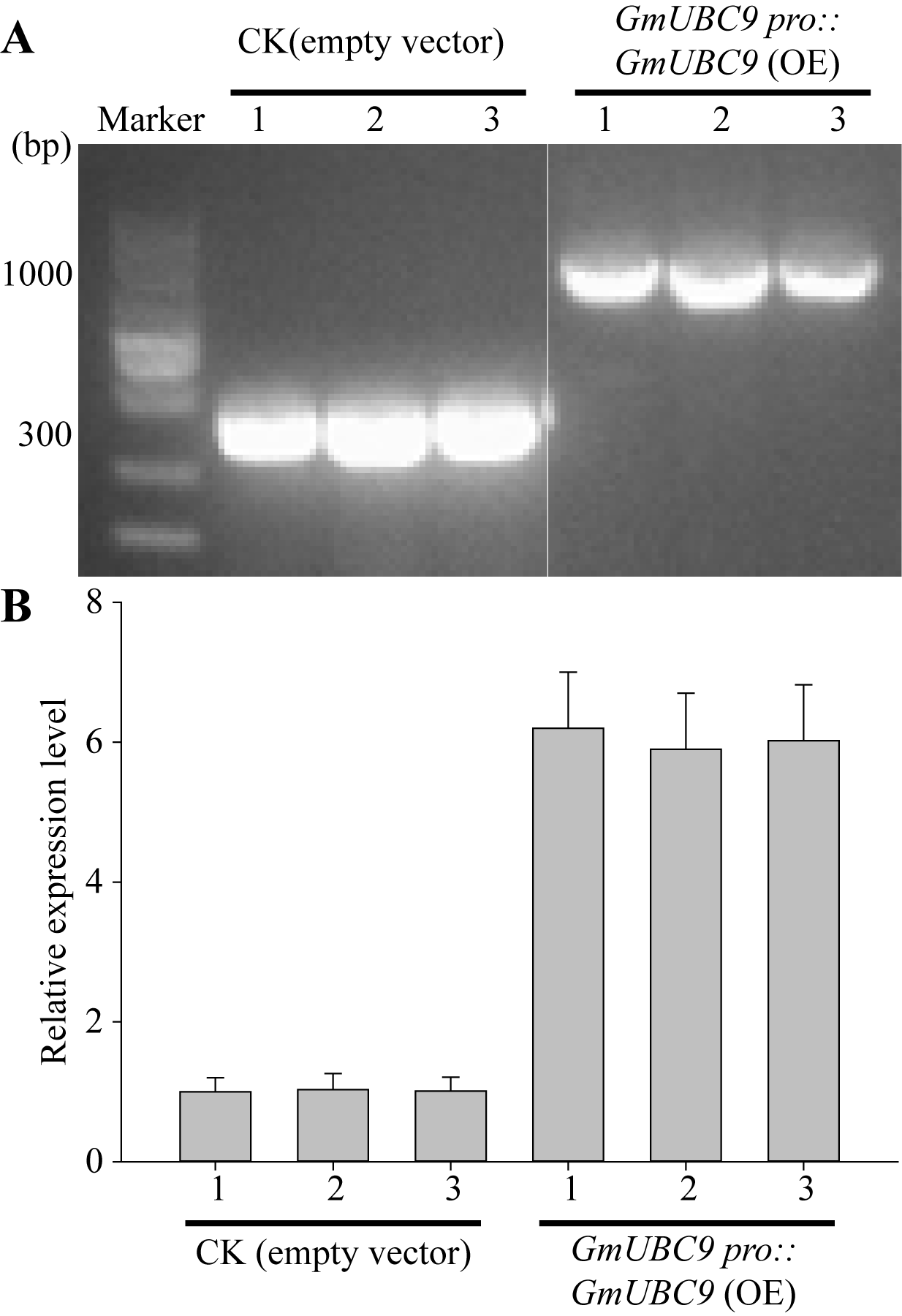

Supplement: Figure S6 — Detection of GmUBC9 in CK (empty vector) and transgenic soybean hairy root lines. (A) Electrophoresis results of amplification products of vector sequences (CK lines) and target gene fragments (OE lines). (B) Relative transcript levels of GmUBC9 in CK and transgenic soybean hairy root lines determined by RT-qPCR. The analysis was performed with 3 independent technical replicates of each transformed root lines. Error bars indicate the SD of three replicates. [file Image_6.tif]

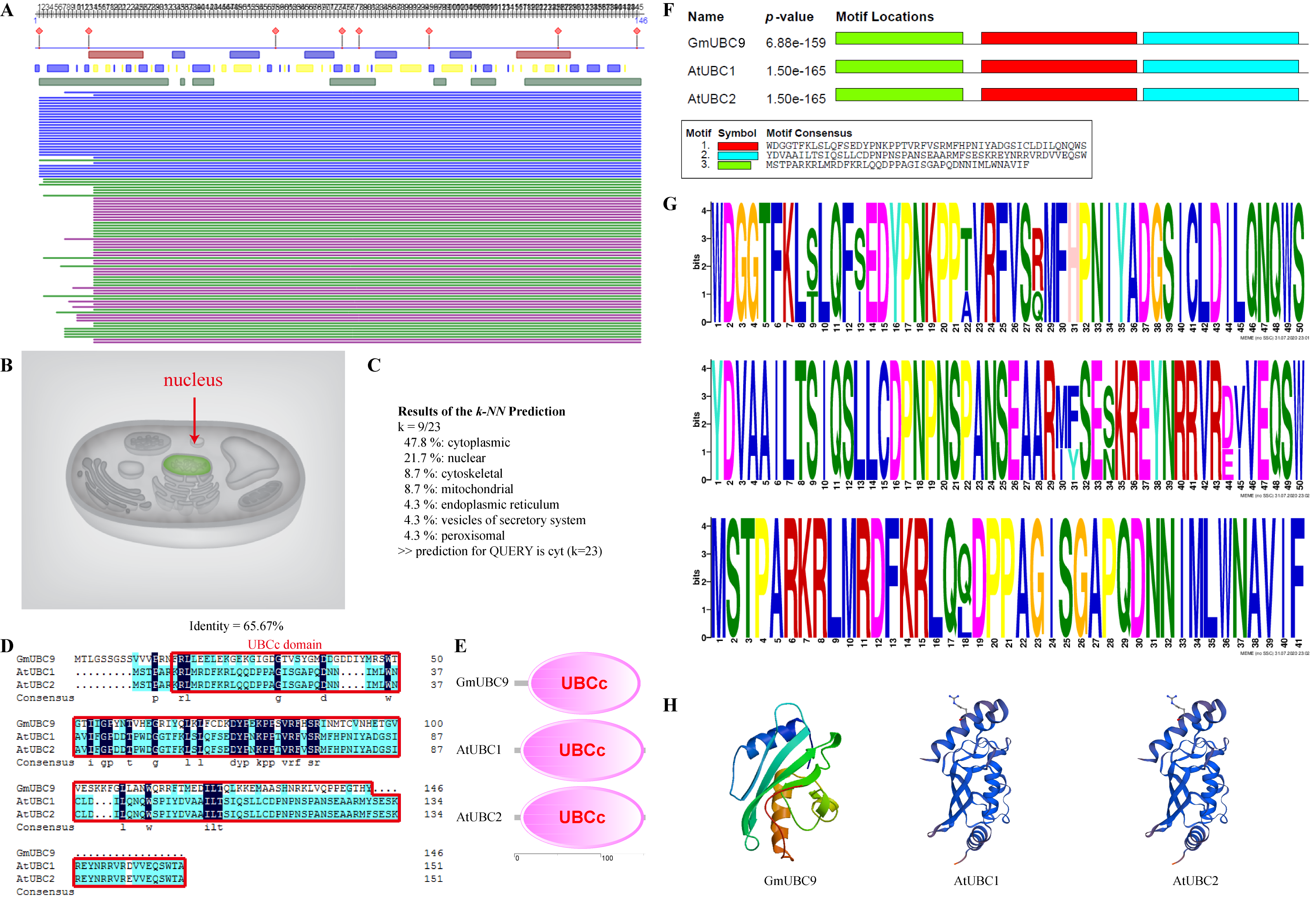

Supplement: Figure S7 — Prediction of GmUBC9 signal peptides and nuclear localization signals (NLS). (A) The red curve represents the probability of a signal peptide (SP-Sec/SPI). Signalp-5.0 software was used for the prediction (http://www.cbs.dtu.dk/services/SignalP/). (B–C) The NLS prediction of GmOXR17. (B) This image was produced using UniProt (https://www.uniprot.org/). The red arrow points to the nucleus. (C) The subcellular localization prediction by PSORT II (http://www.genscript.com/tools/psort). We used the k-NN prediction. The values represent the distribution ratio of GmUBC9 in each subcellular compartment or organelle. (D–F) Homology analysis of GmUBC9 and AtUBC1/2. (D) Alignment of the amino acid sequences of GmUBC9 and AtUBC1/2. Identical residues are shown on a black background. Red box points to the conserved domains of the UBCc domains. (E) Classification of the three proteins based on their domain features. The UBCc representative protein structure for each one was shown. The bar represents 100 amino acids. (F) The predicted domain structures. The predicted structures of the UBCs were analyzed by SMART (http://smart.embl-heidelberg.de/) and were drawn online (https://swissmodel.expasy.org/). [file Image_7.tif]

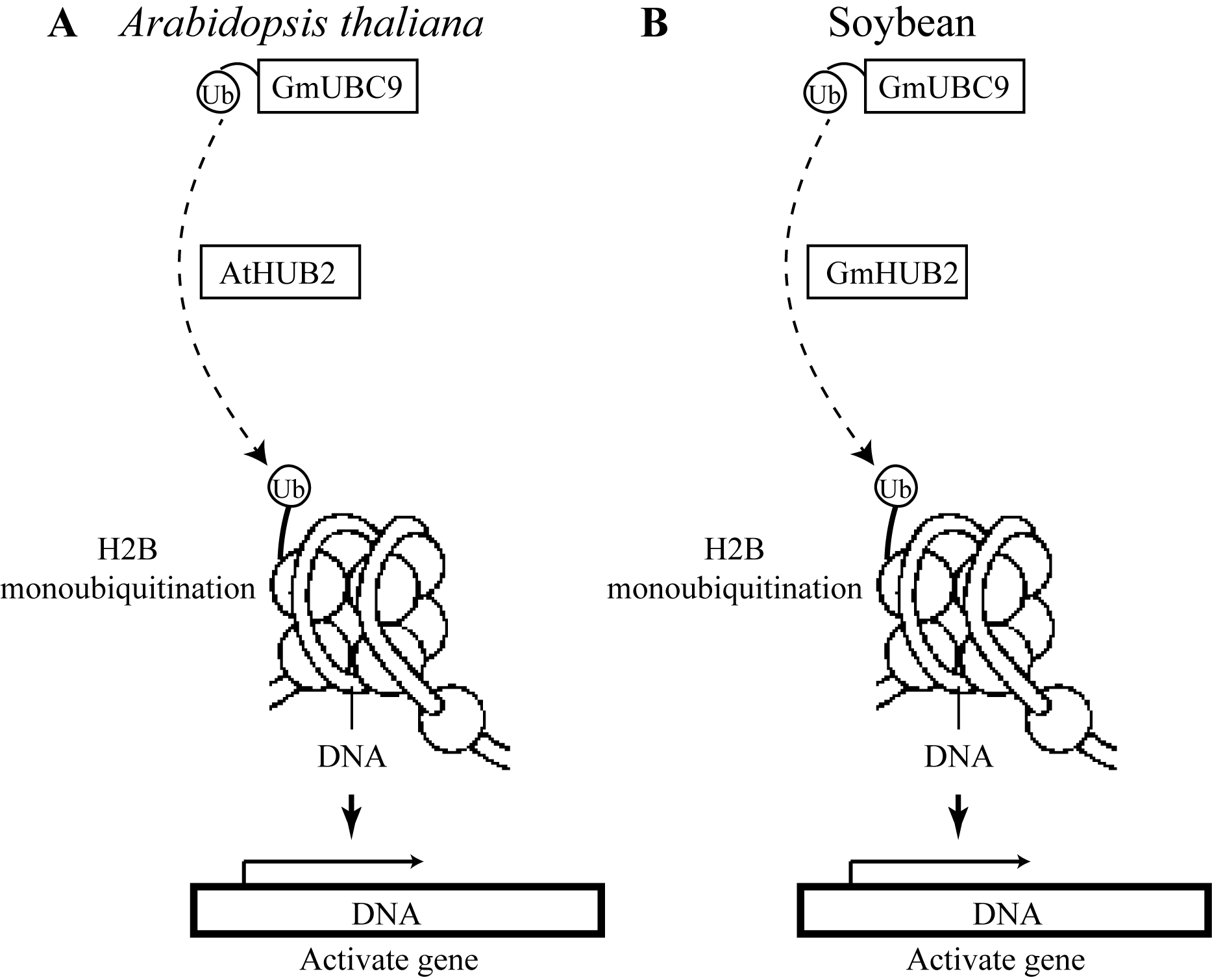

Supplement: Figure S8 — Working model for GmUBC9 in the regulation of stress-responsive and flowering genes in Arabidopsis (A) and soybean (B). H2Bub1 mediated by the GmUBC9 and AtHUB2/GmHUB2 complex plays pivotal roles in stress responses and regulation of flowering. GmUBC9 can interact with AtHUB2/GmHUB2 when plants are exposed to drought stress conditions and activate stress responsive genes by increasing H2Bub1 levels (Figure 10). In addition, the overexpression of GmUBC9 will also increase the level of H2Bub1 and deactivate flowering (Figure 12A, C, E & G). An increase in H2Bub1 level will further activate the expression of downstream stress response genes and FLC family genes (Figure 11; Figure 12F, H) (Cao et al., 2008; Chen et al., 2019). [file Image_8.tif]

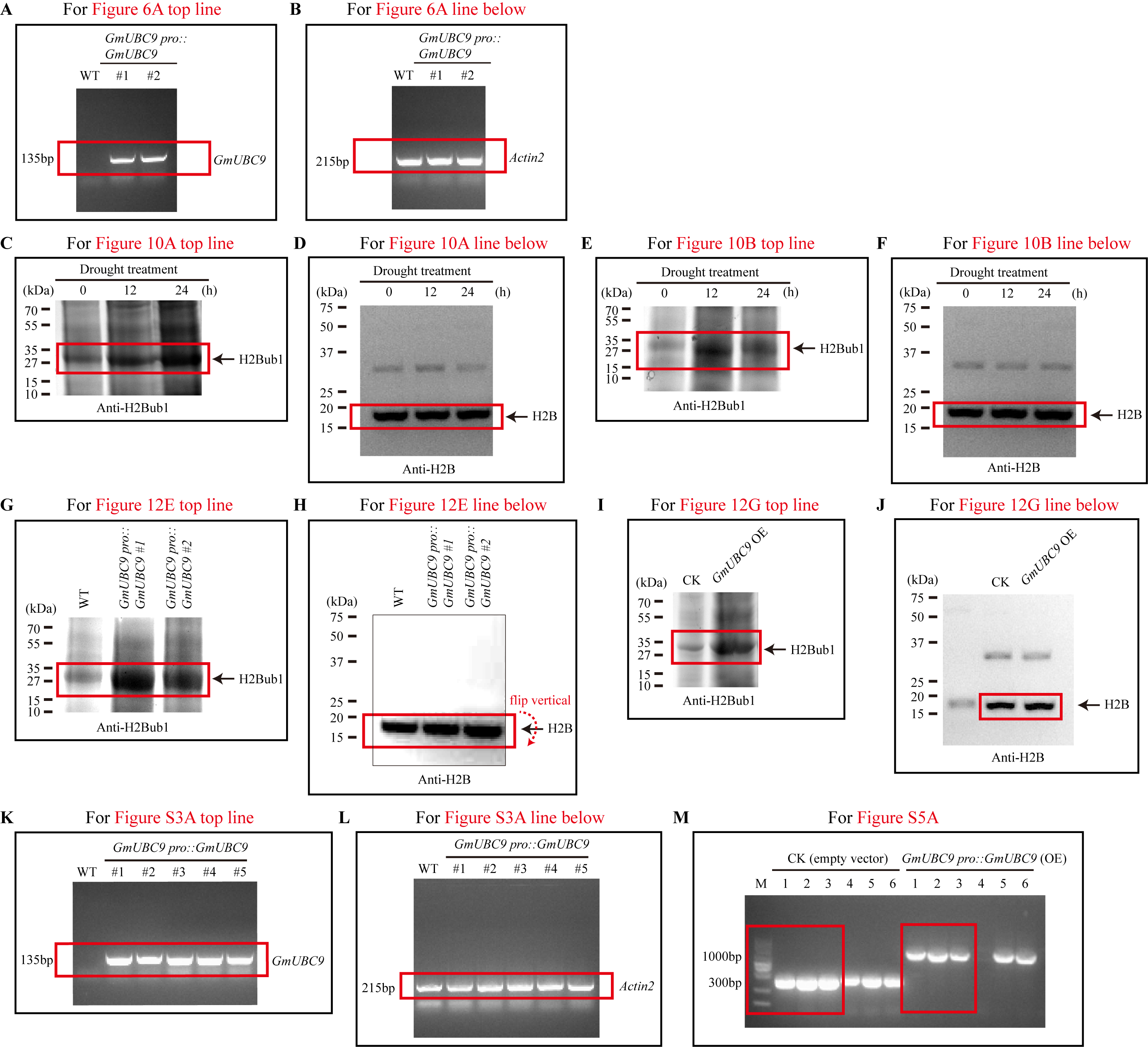

Supplement: Figure S9 — All original images of gel and Western blot. [file Image_9.tif]
