# Supplementary material for: Overexpression of GmUBC9 Gene Enhances Plant Drought Resistance and Affects Flowering Time via Histone H2B Monoubiquitination
Source: Front Plant Sci. 2020 Sep 4;11:555794. doi: 10.3389/fpls.2020.555794 (PMC7498670; doi:10.3389/fpls.2020.555794)
Supplement: Table S1 — Detailed information of the ubiquitin-conjugating enzyme (UBC) genes identified in the soybean genome. [file Table_1.docx]

**TABLE S1 |** Detailed information of the ubiquitin-conjugating enzyme (*UBC*) genes identified in the soybean genome.

| **Number** | **Gene ID number** | **Gene name** | **Amino acid residues** | **MW** | **p*I*** | **Chromosome location** | **UBCc domain** | [**E-value**](http://smart.embl-heidelberg.de/smart/show_motifs.pl) |
| --- | --- | --- | --- | --- | --- | --- | --- | --- |
| 1 | Glyma.01G043600 | *GmUBC1* | 152 | 17255.50 | 5.07 | Chr01:4751170..4754842 | 7-150 | 1.35e-65 |
| 2 | Glyma.01G169700 | *GmUBC2* | 161 | 18432.91 | 7.72 | Chr01:50715084..50719427 | 18-161 | 3.9e-48 |
| 3 | Glyma.02G02000 | *GmUBC3* | 152 | 17291.51 | 5.07 | Chr02:1824031..1827387 | 7-150 | 3.03e-68 |
| 4 | Glyma.02G199700 | *GmUBC4* | 190 | 21879.24 | 9.17 | Chr02:38305652..38309940 | 16-165 | 5.38e-10 |
| 5 | Glyma.02G209800 | *GmUBC5* | 105 | 11697.52 | 5.73 | Chr02:39495534..39496914 |  |  |
| 6 | Glyma.02G236900 | *GmUBC6* | 148 | 16462.04 | 7.72 | Chr02:42488913..42493292 | 4-147 | 4.24e-81 |
| 7 | Glyma.03G004200 | *GmUBC7* | 258 | 27820.73 | 9.07 | Chr03:370538..374055 | 13-=156 | 8.74e-51 |
| 8 | Glyma.03G113100 | *GmUBC8* | 108 | 12662.22 | 5.33 | Chr03:31997439..31998399 | 4-108 | 1.24 |
| 9 | Glyma.03G199900 | *GmUBC9* | 146 | 16611.88 | 6.96 | Chr03:40880573..40884921 | 16-146 | 0.000115 |
| 10 | Glyma.03G255400 | *GmUBC10* | 148 | 16548.13 | 7.72 | Chr03:45047131..45050785 | 4-147 | 1.38e-80 |
| 11 | Glyma.04G173300 | *GmUBC11* | 152 | 17348.56 | 5.37 | Chr04:43367265..43372330 | 7-150 | 4.06e-69 |
| 12 | Glyma.04G199200 | *GmUBC12* | 192 | 21120.23 | 4.80 | Chr04:47137301..47140936 | 5-150 | 1.59e-62 |
| 13 | Glyma.04G238800 | *GmUBC13* | 148 | 16561.13 | 7.72 | Chr04:50751900..50755419 | 4-147 | 9.24e-82 |
| 14 | Glyma.05G028800 | *GmUBC14* | 152 | 17348.56 | 5.37 | Chr05:2476686..2482768 | 7-150 | 4.06e-69 |
| 15 | Glyma.05G035700 | *GmUBC15* | 185 | 20897.28 | 4.31 | Chr05:3138855..3145745 | 4-148 | 1.06e-49 |
| 16 | Glyma.05G140200 | *GmUBC16* | 450 | 50264.54 | 4.65 | Chr05:33281198..33285862 | 172-329 | 5.71e-23 |
| 17 | Glyma.05G209400 | *GmUBC17* | 183 | 20632.99 | 4.27 | Chr05:39137625..39140988 | 4-148 | 5.3e-50 |
| 18 | Glyma.05G235000 | *GmUBC18* | 159 | 17922.49 | 4.96 | Chr05:41182783..41188063 | 14-158 | 1.28e-16 |
| 19 | Glyma.06G124900 | *GmUBC19* | 148 | 16561.13 | 7.72 | Chr06:10168532..10171636 | 4-147 | 9.24e-82 |
| 20 | Glyma.06G166300 | *GmUBC20* | 192 | 21119.21 | 4.87 | Chr06:13859494..13862957 | 5-150 | 5.62e-63 |
| 21 | Glyma.06G190900 | *GmUBC21* | 116 | 13298.96 | 4.67 | Chr06:16769976..16773415 | 1-114 | 5.86e-43 |
| 22 | Glyma.06G230300 | *GmUBC22* | 153 | 17191.82 | 6.14 | Chr06:35837790..35843235 | 8-151 | 4.93e-68 |
| 23 | Glyma.06G243300 | *GmUBC23* | 869 | 92699.06 | 5.36 | Chr06:40488801..40495206 | 493-650 | 1.75e-18 |
| 24 | Glyma.07G046200 | *GmUBC24* | 238 | 27112.31 | 9.25 | Chr07:3879069..3883801 | 8-162 | 5.19e-35 |
| 25 | Glyma.07G069300 | *GmUBC25* | 183 | 20952.45 | 4.44 | Chr07:6262886..6265779 | 4-148 | 1.09e-51 |
| 26 | Glyma.07G168100 | *GmUBC26* | 159 | 18119.65 | 5.27 | Chr07:27445431..27453697 | 14-158 | 3.02e-13 |
| 27 | Glyma.07G196500 | *GmUBC27* | 927 | 104379.50 | 5.13 | Chr07:36483372..36490967 | 681-838 | 4.7e-33 |
| 28 | Glyma.07G239100 | *GmUBC28* | 324 | 37156.36 | 9.65 | Chr07:42013261..42014235 | 39-193 | 1.87e-27 |
| 29 | Glyma.07G240600 | *GmUBC29* | 309 | 34433.99 | 5.24 | Chr07:42119013..42121553 | 13-173 | 2.3e-27 |
| 30 | Glyma.08G016100 | *GmUBC30* | 183 | 20621.98 | 4.26 | Chr08:1271249..1274468 | 4-148 | 1.22e-49 |
| 31 | Glyma.08G042500 | *GmUBC31* | 159 | 17938.55 | 4.96 | Chr08:3356622..3361114 | 14-158 | 7.9e-17 |
| 32 | Glyma.08G095600 | *GmUBC32* | 474 | 53028.58 | 4.70 | Chr08:7278090..7282776 | 194-351 | 4.57e-22 |
| 33 | Glyma.08G113700 | *GmUBC33* | 176 | 19227.83 | 6.05 | Chr08:8723285..8726038 | 33-176 | 3.14e-52 |
| 34 | Glyma.08G267400 | *GmUBC34* | 148 | 16411.90 | 6.88 | Chr08:33557970..33560582 | 4-147 | 1.54e-78 |
| 35 | Glyma.08G297300 | *GmUBC35* | 152 | 17320.55 | 5.37 | Chr08:41348459..41357671 | 7-150 | 3.54e-69 |
| 36 | Glyma.09G036300 | *GmUBC36* | 308 | 34552.00 | 5.20 | Chr09:3034310..3036945 | 13-173 | 5.08e-29 |
| 37 | Glyma.09G124800 | *GmUBC37* | 159 | 17927.44 | 5.58 | Chr09:30448338..30454983 | 14-158 | 3.83e-15 |
| 38 | Glyma.09G257800 | *GmUBC38* | 185 | 21079.49 | 4.32 | Chr09:47667497..47670973 | 9-150 | 1.58e-48 |
| 39 | Glyma.09G273100 | *GmUBC39* | 148 | 16476.06 | 7.72 | Chr09:48941211..48945532 | 4-147 | 2.44e-81 |
| 40 | Glyma.10G077600 | *GmUBC40* | 146 | 16614.93 | 6.20 | Chr10:8567994..8571174 | 16-144 | 0.000146 |
| 41 | Glyma.10G096200 | *GmUBC41* | 148 | 16541.95 | 6.39 | Chr10:14930665..14936448 | 4-147 | 9.6e-80 |
| 42 | Glyma.10G212200 | *GmUBC42* | 186 | 20758.42 | 4.84 | Chr10:44476625..44479276 | 40-183 | 3.31e-51 |
| 43 | Glyma.10G282000 | *GmUBC43* | 1128 | 124205.20 | 4.66 | Chr10:50294239..50301335 | 880-1037 | 4.26e-22 |
| 44 | Glyma.11G073600 | *GmUBC44* | 161 | 18432.91 | 7.72 | Chr11:5491229..5495573 | 18-161 | 3.9e-48 |
| 45 | Glyma.11G095700 | *GmUBC45* | 181 | 20052.92 | 5.49 | Chr11:7263413..7265368 | 39-178 | 4.69e-42 |
| 46 | Glyma.11G107800 | *GmUBC46* | 148 | 16528.08 | 7.68 | Chr11:8229860..8231508 | 4-147 | 3.19e-76 |
| 47 | Glyma.11G140900 | *GmUBC47* | 166 | 18600.04 | 5.05 | Chr11:10763326..10769634 | 7-164 | 5.31e-73 |
| 48 | Glyma.11G199400 | *GmUBC48* | 148 | 16504.07 | 7.72 | Chr11:27757011..27761038 | 4-147 | 1.56e-79 |
| 49 | Glyma.12G021800 | *GmUBC49* | 180 | 20191.77 | 5.54 | Chr12:1572976..1574844 | 39-180 | 8.72e-60 |
| 50 | Glyma.12G032800 | *GmUBC50* | 148 | 16498.07 | 7.68 | Chr12:2479542..2481426 | 4-147 | 3.78e-78 |
| 51 | Glyma.12G064400 | *GmUBC51* | 167 | 18671.12 | 5.05 | Chr12:4746377..4751917 | 8-165 | 5.31e-73 |
| 52 | Glyma.12G153900 | *GmUBC52* | 389 | 42785.28 | 6.05 | Chr12:23572762..23575150 | 92-249 | 8.08e-23 |
| 53 | Glyma.12G154000 | *GmUBC53* | 304 | 33163.95 | 6.16 | Chr12:23616912..23619436 | 7-164 | 4.36e-19 |
| 54 | Glyma.12G161200 | *GmUBC54* | 153 | 17247.93 | 6.74 | Chr12:29673720..29680486 | 8-151 | 3.48e-68 |
| 55 | Glyma.12G224200 | *GmUBC55* | 704 | 78510.16 | 5.92 | Chr12:38352836..38359778 | 443-600 | 7.25e-25 |
| 56 | Glyma.12G228900 | *GmUBC56* | 151 | 17433.56 | 9.59 | Chr12:38907362..38909342 | 8-142 | 6.69e-38 |
| 57 | Glyma.13G00700 | *GmUBC57* | 64 | 7531.67 | 7.80 | Chr13:1960877..1961591 |  |  |
| 58 | Glyma.13G040500 | *GmUBC58* | 149 | 16673.26 | 7.72 | Chr13:12904066..12907723 | 4-148 | 2.44e-81 |
| 59 | Glyma.13G179600 | *GmUBC59* | 933 | 104609.77 | 5.00 | Chr13:29318815..29323855 | 680-837 | 1.6e-35 |
| 60 | Glyma.13G239100 | *GmUBC60* | 919 | 102807.79 | 5.09 | Chr13:34957148..34964627 | 673-830 | 1.55e-33 |
| 61 | Glyma.13G270800 | *GmUBC61* | 153 | 17205.85 | 6.74 | Chr13:37275486..37279668 | 8-151 | 2.83e-68 |
| 62 | Glyma.13G277400 | *GmUBC62* | 723 | 80513.60 | 6.09 | Chr13:37872791..37879361 | 462-619 | 6.56e-23 |
| 63 | Glyma.13G334800 | *GmUBC63* | 166 | 18610.08 | 5.05 | Chr13:42840520..42845919 | 7-164 | 2.85e-73 |
| 64 | Glyma.14G124300 | *GmUBC64* | 148 | 16627.19 | 7.73 | Chr14:18984437..18988305 | 4-147 | 3.45e-81 |
| 65 | Glyma.14G205700 | *GmUBC65* | 148 | 16462.04 | 7.72 | Chr14:47085767..47091016 | 4-147 | 4.24e-81 |
| 66 | Glyma.15G039700 | *GmUBC66* | 166 | 18610.08 | 5.05 | Chr15:3130909..3135828 | 7-164 | 2.85e-73 |
| 67 | Glyma.15G074200 | *GmUBC67* | 924 | 103642.60 | 5.00 | Chr15:5671022..5678312 | 678-835 | 1.2e-30 |
| 68 | Glyma.15G141200 | *GmUBC68* | 306 | 34309.72 | 5.32 | Chr15:11557041..11559982 | 13-173 | 2.96e-30 |
| 69 | Glyma.15G189500 | *GmUBC69* | 159 | 18527.31 | 8.95 | Chr15:20105256..20107586 | 7-155 | 4.48e-7 |
| 70 | Glyma.16G014400 | *GmUBC70* | 238 | 27092.35 | 9.24 | Chr16:1263516..1268395 | 8-162 | 3.67e-35 |
| 71 | Glyma.16G035000 | *GmUBC71* | 183 | 20938.42 | 4.44 | Chr16:3311992..3314979 | 4-148 | 1.09e-51 |
| 72 | Glyma.16G102600 | *GmUBC72* | 82 | 9202.59 | 7.96 | Chr16:20800599..20800913 | 1-81 | 1.23e-13 |
| 73 | Glyma.16G102700 | *GmUBC73* | 80 | 8966.45 | 9.21 | Chr16:20801555..20802705 | 4-79 | 0.0054 |
| 74 | Glyma.16G103100 | *GmUBC74* | 148 | 16469.03 | 8.44 | Chr16:20857482..20859594 | 4-147 | 5.19e-76 |
| 75 | Glyma.16G103200 | *GmUBC75* | 82 | 9229.66 | 8.68 | Chr16:20889936..20890250 | 1-81 | 8.54e-13 |
| 76 | Glyma.17G032800 | *GmUBC76* | 311 | 34778.29 | 5.40 | Chr17:2405236..2407790 | 13-173 | 4.06e-28 |
| 77 | Glyma.17G034200 | *GmUBC77* | 338 | 38913.39 | 6.02 | Chr17:2496617..2497633 | 48-205 | 6.31e-25 |
| 78 | Glyma.17G034400 | *GmUBC78* | 333 | 38263.84 | 9.30 | Chr17:2513264..2514265 | 41-193 | 9.64e-21 |
| 79 | Glyma.17G091700 | *GmUBC79* | 184 | 20807.23 | 4.38 | Chr17:7137499..7144510 | 4-148 | 3.63e-48 |
| 80 | Glyma.17G098000 | *GmUBC80* | 152 | 17360.62 | 5.37 | Chr17:7720344..7724128 | 7-150 | 1.23e-68 |
| 81 | Glyma.18G051400 | *GmUBC81* | 148 | 16446.03 | 7.71 | Chr18:4433782..4438576 | 4-147 | 8.82e-79 |
| 82 | Glyma.18G124600 | *GmUBC82* | 131 | 14950.47 | 7.79 | Chr18:16239780..16241762 | 7-127 | 5.74e-37 |
| 83 | Glyma.18G216000 | *GmUBC83* | 158 | 17587.34 | 7.67 | Chr18:50303953..50307600 | 4-157 | 2.82e-77 |
| 84 | Glyma.18G234800 | *GmUBC84* | 183 | 21045.59 | 4.40 | Chr18:52319630..52322552 | 4-148 | 6.27e-52 |
| 85 | Glyma.19G072800 | *GmUBC85* | 266 | 28860.05 | 9.12 | Chr19:25957072..25962027 | 13-156 | 3.63e-48 |
| 86 | Glyma.19G121700 | *GmUBC86* | 259 | 27973.99 | 9.07 | Chr19:37997514..38001601 | 13-156 | 9.51e-52 |
| 87 | Glyma.19G197500 | *GmUBC87* | 170 | 19477.35 | 6.51 | Chr19:45477651..45482134 | 16-165 | 1.87e-9 |
| 88 | Glyma.19G253000 | *GmUBC88* | 148 | 16548.13 | 7.72 | Chr19:49838828..49842550 | 4-147 | 1.38e-80 |
| 89 | Glyma.20G038400 | *GmUBC89* | 167 | 18874.65 | 9.25 | Chr20:5969852..5974456 | 64-166 | 1.35e-24 |
| 90 | Glyma.20G107300 | *GmUBC90* | 1124 | 123662.38 | 4.61 | Chr20:34991244..34999277 | 876-1033 | 4.26e-22 |
| 91 | Glyma.20G178900 | *GmUBC91* | 186 | 20929.68 | 4.95 | Chr20:41626790..41629619 | 40-185 | 1.54e-51 |
