# Supplementary material for: Overexpression of GmUBC9 Gene Enhances Plant Drought Resistance and Affects Flowering Time via Histone H2B Monoubiquitination
Source: Front Plant Sci. 2020 Sep 4;11:555794. doi: 10.3389/fpls.2020.555794 (PMC7498670; doi:10.3389/fpls.2020.555794)
Supplement: Table S2 — Primary cis-acting elements in the soybean Class I UBC gene promoter regions. ABRE: ABA-responsive element; ARE: anaerobic-responsive element; DRE: drought-responsive element; LTR: low-temperature responsive element; MYB: response to drought and ABA signals; MYC: response to drought, ABA and cold signals; TC-rich repeat: defense and stress responsiveness. [file Table_2.docx]

**TABLE S2 |** Main cis-acting elements in the promoter regions of soybean Class Ⅰ *UBC* genes. ABRE: ABA-responsive element; ARE: anaerobic-responsive element; DRE: drought-responsive element; LTR: low-temperature responsive element; MYB: response to drought and ABA signals; MYC: response to drought, ABA and cold signals; TC-rich repeat: defense and stress responsiveness.

|  | **ABRE** | **ARE** | **DRE** | **LTR** | **MYB** | **MYC** | **TC-rich repeats** |
| --- | --- | --- | --- | --- | --- | --- | --- |
| *GmUBC4* | 1 | 2 | 0 | 0 | 4 | 3 | 0 |
| *GmUBC5* | 0 | 3 | 1 | 1 | 4 | 1 | 0 |
| *GmUBC8* | 0 | 3 | 1 | 1 | 10 | 5 | 0 |
| *GmUBC9* | 3 | 1 | 2 | 0 | 10 | 4 | 1 |
| *GmUBC18* | 0 | 1 | 0 | 0 | 9 | 5 | 0 |
| *GmUBC26* | 11 | 3 | 0 | 1 | 12 | 7 | 0 |
| *GmUBC31* | 3 | 0 | 0 | 0 | 6 | 6 | 1 |
| *GmUBC37* | 5 | 2 | 0 | 0 | 16 | 8 | 1 |
| *GmUBC40* | 0 | 2 | 0 | 0 | 3 | 8 | 0 |
| *GmUBC69* | 7 | 1 | 0 | 2 | 10 | 7 | 0 |
| *GmUBC87* | 0 | 1 | 0 | 0 | 8 | 4 | 0 |
