# Supplementary material for: Overexpression of GmUBC9 Gene Enhances Plant Drought Resistance and Affects Flowering Time via Histone H2B Monoubiquitination
Source: Front Plant Sci. 2020 Sep 4;11:555794. doi: 10.3389/fpls.2020.555794 (PMC7498670; doi:10.3389/fpls.2020.555794)
Supplement: Table S3 — Pearson correlated expression analysis of GmUBC9. The annotation of correlated genes (an important part of it) (Supporting FIGURE S4) has been shown. Protein sequence and functional comments came from Phytozome site (https://phytozome.jgi.doe.gov/pz/portal.html#). Subcellular localization prediction was performed using PSORT II Prediction (https://psort.hgc.jp/form2.html) and Predict Protein (https://www.predictprotein.org/home). [file Table_3.docx]

**TABLE S3 |** Pearson correlated expression analysis of *GmUBC9*. The annotation of correlated genes (an important part of it) (**support** **FIGURE S4**). Protein sequence and functional comments came from Phytozome site (https://phytozome.jgi.doe.gov/pz/portal.html#). Subcellular localization prediction was performed at sites PSORT II Prediction (https://psort.hgc.jp/form2.html) and Predict Protein (https://www.predictprotein.org/home).

| Number | Gene ID | The protein sequence | Results of the k-NN Prediction by PSORT II Prediction | Results of the prediction by Predict Protein | Annotation |
| --- | --- | --- | --- | --- | --- |
| 1 | Glyma.01G154400.1 | >MPQNCIAPKPEFCNSHNSVEGPPEMTEPHNSTVLSYPMQANEQQQQPFPKLIMYPITLKFEELVYKVKLEQKGGCWGSTWTCKEKTILNGITGVVCPGEILAMLGPSGSGKTTLLTALGGRLNGKLSGKITYNGQPFSGAMKRRTGFVAQDDVLYPHLTVTETLVFTALLRLPNTLKRDEKVQHVERVITELGLTRCRSSMIGGPLFRGISGGEKKRVSIGQEMLINPSLLLLDEPTSGLDSTTAQRILNTIKRLASGGRTVVTTIHQPSSRLYYMFDKVVLLSEGCPIYYGPASTALDYFSSVGFSTCVTVNPADLLLDLANGIAPDSKHATEQSEGLEQERKQVRESLISAYEKNIATRLKAEVCSLEANNYNITKDACARNSIKPDQWCTSWWHQFKVLLQRGVRERRYEAFNRLRIFQVVSVAFLGGLLWWHTPESHIDDRVALLFFFSVFWGFYPLYNAVFTFPQERRMLIKERSSGMYRLSSYFLARTIGDLPLELALPTAFVFIIYWMGGLKPDPMTFILSLLVVLYSVVVSQSLGLAFGAILMEVKQATTLASVTTLVFLIAGGYYIQQIPPFIVWLKYLSYSYYCYKLLLGVQYNENDYYECSKEELCKVADFPPIKSMGLNHLWVDVCIMAMMLVGYRLVAYLALHRVR | *k* = 9/23  52.2 %: plasma membrane  26.1 %: endoplasmic reticulum  8.7 %: nuclear  8.7 %: mitochondrial  4.3 %: vesicles of secretory system  >> prediction for QUERY is pla (k=23) | Predicted localization for the Eukarya domain: Plasma Membrane (GO term ID: [GO:0005886](http://amigo.geneontology.org/cgi-bin/amigo/term_details?term=GO:0005886)) Prediction confidence 18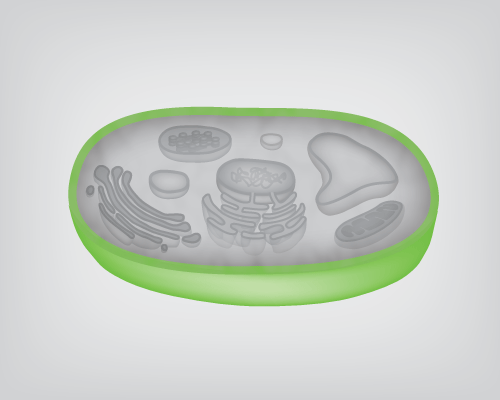 | (1 of 5) PTHR19241:SF213 - ABC TRANSPORTER G FAMILY MEMBER 14 |
| 2 | Glyma.07G119100.1 | >MAEEESPSVMPKVITFLSSLLERVAESNDHNQQHQKISVFHGLTRPNISIHSYLERIFKYANCSPSCFVVAYVYLDRFTQRQPSLPINTFNVHRLLITSVMVAAKFMDDMYYNNAYYAKVGGITKIEMNFLELDFLFGLGFHLNVTPGTFQAYCVNLQREMLLIQQPLNFADSTLNLGKSLKAHLCFNEDESSHQKQQQLAV | k = 9/23  60.9 %: cytoplasmic  13.0 %: nuclear  4.3 %: Golgi  4.3 %: cytoskeletal  4.3 %: vacuolar  4.3 %: endoplasmic reticulum  4.3 %: vesicles of secretory system  4.3 %: mitochondrial  >> prediction for QUERY is cyt (k=23) | Predicted localization for the Eukarya domain: Cytoplasm (GO term ID: GO:0005737) Prediction confidence 25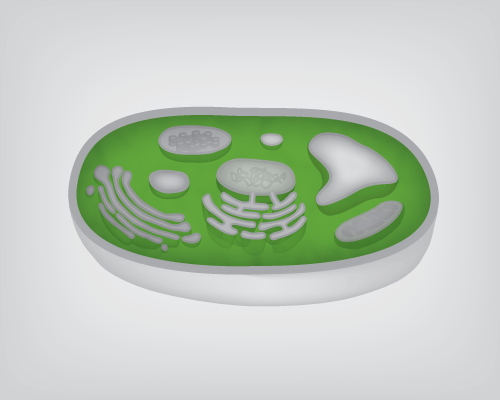 | (1 of 6) PTHR15615:SF28 - CYCLIN-U4-1 |
| 3 | Glyma.09G250300.1 | >MAFVTTAEVCDANPQLILSGELRALQPVFQIYGRRQVFSGPIVTLKVFEDNVLVREFLEEKGNGRVLVVDGGASLRCAILGGNPVVQAQNNGWAGIVVNGCIRDVDEINGCDIGVRALASHPMKANKKGMGEKHVPVNIAGTRISDGDWLYADTDGILISRTELSV | k = 9/23  60.9 %: cytoplasmic  17.4 %: nuclear  13.0 %: mitochondrial  4.3 %: vesicles of secretory system  4.3 %: vacuolar  >> prediction for QUERY is cyt (k=23) | Predicted localization for the Eukarya domain: Cytoplasm (GO term ID: GO:0005737) Prediction confidence 24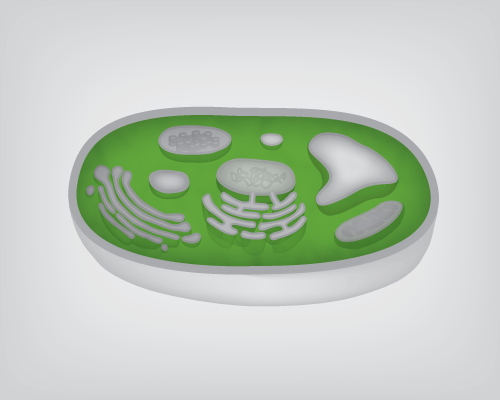 | (1 of 4) 4.1.1.3 - Oxaloacetate decarboxylase / Oxaloacetate carboxy-lyase |
| 4 | Glyma.13G306700.1 | >MATGGDGDTSKQELFQLIKRFGAYVTFKISNLFPLSLHNLDLRSIGAVAGLAVAIVFTWRLLRSPSGSQRRQQKRQGPSSSNPGVGTNSNSNASVVPSDACSPSDDSRAQNVVDEFFQPVKPTLGQIVRQKLSEGRKVTCRLLGVILEESSPEELQKQATVRSSVLEVLLEVTKFCDLYLMERVLDDESEKRVLVALEEAGVFTSGGLVKDKVLFCSTENGRSSFVRQLEPDWHIDSNPEIVTQLARFIKYQLHVSPYKTERTAANVFSAPSLEQFFGSI | k = 9/23  39.1 %: cytoplasmic  21.7 %: nuclear  17.4 %: mitochondrial  8.7 %: endoplasmic reticulum  4.3 %: Golgi  4.3 %: peroxisomal  4.3 %: vesicles of secretory system  >> prediction for QUERY is cyt (k=23) | Predicted localization for the Eukarya domain: Nucleus (GO term ID: GO:0005634) Prediction confidence 27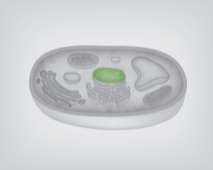 | (1 of 2) PTHR34126:SF1 - PEROXISOME BIOGENESIS PROTEIN 22 |
| 5 | Glyma.20G054900.1 | >MPHKAKHSKKRGSHGISNLVAHASSQAQSYAPTLSHIPSSIPTTLDPLSRSLLPQNVFSSILNLICQAAYQGYSMLEELPQHSSKLAQPFAPTITDIRPSIPIAQDLVSQSNIPSCSMLRKSKRSKKRVSHAISNLMPHASSQGHSKPMELPLQPSELAQPCAPPVVLVPSSISVTQDPVLPSDPSSIPVNQDPVLPSDPSSIPVNQDLVSPSDSSSIPANQDPSSIAVNQDPVSPSDSSSIPVNQDPVSPSDPSSSPILKI | k = 9/23  52.2 %: mitochondrial  39.1 %: nuclear  8.7 %: cytoskeletal  >> prediction for QUERY is mit (k=23) | Predicted localization for the Eukarya domain: Plasma Membrane (GO term ID: GO:0005886) Prediction confidence 8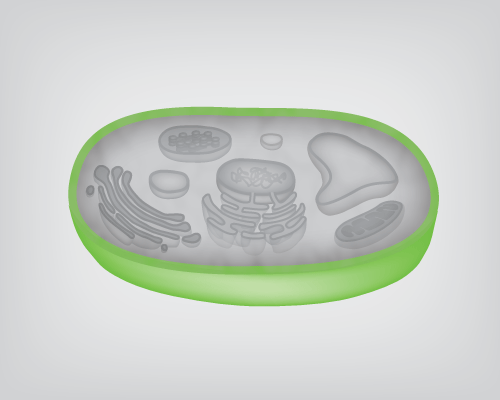 | (1 of 2) PTHR33144:SF3 - PLANT TRANSPOSASE (PTTA/EN/SPM FAMILY) |
