# Supplementary material for: Overexpression of GmUBC9 Gene Enhances Plant Drought Resistance and Affects Flowering Time via Histone H2B Monoubiquitination
Source: Front Plant Sci. 2020 Sep 4;11:555794. doi: 10.3389/fpls.2020.555794 (PMC7498670; doi:10.3389/fpls.2020.555794)
Supplement: Table S4 — Primers used in this study. [file Table_4.docx]

**TABLE S4 |** Primers used in this study.

| **Names** | **Sequences** |
| --- | --- |
| **Primers used for quantitative RT-PCR** | |
| *GmUBC4* | 5’-TGAAGAAGGAAATGGCCGCT-3’ |
|  | 5’-GGCGTATGTATCCTCCAGGC-3’ |
| *GmUBC5* | 5’-TAGAGTGTGCACATGCAATT-3’ |
|  | 5’-CAGCACCAAATGCATAAAGC-3’ |
| *GmUBC8* | 5’-GCTGAAGGAGATTGAACGTGG-3’ |
|  | 5’-ACACCAGTCATGTTGATCCGT-3’ |
| *GmUBC9* | 5’-TCAGGATCCAGTGTCGTGGT-3’ |
|  | 5’-TGAAACCGAACACTTGGGGG-3’ |
| *GmUBC18* | 5’-AAGGTTCCACTGTCGTCGTTC-3’ |
|  | 5’-TGGCTCAACCACTCCAGTTTC-3’ |
| *GmUBC26* | 5’-ATGGGTTCCGAAGGATCCAG-3’ |
|  | 5’-AACAGTACTAGGGGGACCGA-3’ |
| *GmUBC31* | 5’-GCTTGAGAGAGGTGAGAAGGG-3’ |
|  | 5’-AAATGTGGCTCAACCACTCCA-3’ |
| *GmUBC37* | 5’-TGCAAGAGCTTGAGAGAGGTG-3’ |
|  | 5’-TCAACTGGTGGATACGCCCT-3’ |
| *GmUBC40* | 5’-GAGGAGCTTGAACGTGGAGAA-3’ |
|  | CCGAACACTTGGGGGCTTTT-3’ |
| *GmUBC69* | 5’-AGACTACCCAGAAAAGCCCC-3’ |
|  | 5’-GGTTGGACAAGCTTCCGGTT-3’ |
| *GmUBC87* | 5’-CGAGCGTCCGGTTTCATTCA-3’ |
|  | 5’-TGTGAAGCCGCCATTTCCTT-3’ |
| *GmCYP2* | 5’-CTGTGTCGGTGGCTCTGAAT-3’ |
|  | 5’-CCATAACACCGCAATCCCCT-3’ |
| *AtActin2* | 5’-CTCCCTCACAACAACCGC-3’ |
|  | 5’-TACCAGGAACTTCCATACCAAC-3’ |
| *AtHUB2* | 5’-GAAGCTGCAATTGTGAGGCTC-3’ |
|  | 5’-ATTTCCGGTGTCGGATCTCT-3’ |
| *AtP5CS* | 5’-TGACCAGCTTGATGTGACGG-3’ |
|  | 5’-TTCCAACGCCAGTAGAGCAG-3’ |
| *AtDREB* | 5’-ATTTCCCTCGGTCTGATGCG-3’ |
|  | 5’-TCAGCCAATGCTTATCCGCT-3’ |
| *AtRD22* | 5’-GTCCGGTTTAACGCTGAGGA-3’ |
|  | 5’-TCAGTGGAAACAGCCCTGAC-3’ |
| *AtMKK2* | 5’-GACTGGCCAATTTTTCGCCT-3’ |
|  | 5’-TGCTAGAGATCCTCCGTCCA-3’ |
| *GmHUB2* | 5’-ACTCAGACAACGACGAACCC-3’ |
|  | 5’-TCGTCAACCAACTGAGTCCA-3’ |
| *GmP5CS* | 5’-CGCATGCTGGCAGATATGGA-3’ |
|  | 5’-GCATTTGATCGTCGGGCTTC-3’ |
| *GmDREB* | 5’-AAGGAGTACAACAGGCAGCA-3’ |
|  | 5’-TCTGCCTCACACCTCTGAAC-3’ |
| *GmRD22* | 5’-GTACTGTGCCACTTCGCTTG-3’ |
|  | 5’-ACCGCTTTAACCCTAACCCC-3’ |
| *GmMKK2* | 5’-GCTGCCATCTGTAAGCAGGT-3’ |
|  | 5’-TGTAGTTGTAGCCTCGCTGG-3’ |
| *AtFLC* | 5’-CCTCTCCGTGACTAGAGCCAAG-3’ |
|  | 5’-AGGTGACATCTCCATCTCAGCTTC-3’ |
| *AtMAF4* | 5’-GCTTCTCAAGTAACCACCATCAC-3’ |
|  | 5’-CTTGGATGACTTTTCCGTAGCAG-3’ |
| *GmActin* | 5’-GGTGTCATGGTTGGTATGGGTC-3’ |
|  | 5’-CCTCTGTGAGTAGAACTGGGTGC-3’ |
| *GmFLC* | 5’-AACATTCTCCAAGAGGCGCA-3’ |
|  | 5’-CAGTACCGCTCGCTCCTAAA-3’ |
| *GmMAF4* | 5’-TCCGTTCTTTGTGATGCCGA-3’ |
|  | 5’-CCATTACCAAGGCTTCCCGT-3’ |
| *GmTUB* | 5’-TCTTGGACAACGAAGCCATCT-3’ |
|  | 5’-TGGTGAGGGACGAAATGATCT-3’ |
| **Primers used for sequences cloning** | |
| *GmUBC9 pro-*FOR | 5’-TGAATTGAGTTTAATCATCCCCAAA-3’ |
| *GmUBC9 pro-*REV | 5’-AAAAATTAATGTTGGAATAACCAAG-3’ |
| *GmUBC9-*FOR | 5’-ATGACGCTTGGCTCTTCAGG-3’ |
| *GmUBC9-*REV | 5’-CTAATAGTGAGTACCTTCAG-3’ |
| *AtHUB2-*FOR | 5’-ATGGAGAATCAGGAATCGGACGAGCCGATG-3’ |
| *AtHUB2-*REV | 5’-TTACATTTTGACAAGCCGGACGTCATTCTG-3’ |
| *GmHUB2-*FOR | 5’-ATGGAAAACTCAGACAACGACGAACCCGAA-3’ |
| *GmHUB2-*REV | 5’-TCATATTTTTACAAAGCGGACATCACTCTG-3’ |
| **Primers used for the transformation of soybean hairy roots (pCAMBIA3301)** | |
| *GmUBC9* (3301) | 5’-GGACTCTTGACCATGATGACGCTTGGCTCT-3’ |
|  | 5’-ATTCGAGCTGGTCACCCTAATAGTGAGTACC-3’ |
| **Primers used for the transformation of *Arabidopsis* overexpressed lines (pCAMBIA1302)** | |
| *GmUBC9* (1302) | 5’-GGGACTCTTGACCATGATGACGCTTGGCTCT-3’ |
|  | 5’-TCAGATCTACCCATGGATAGTGAGTACCTTCAG-3’ |
| **Primers used for subcellular localization (p16318hGFP)** | |
| *GmUBC9* | 5’-TATCTCTAGAGGATCCATGACGCTTGGCTCT-3’ |
|  | 5’-TGCTCACCATGGATCCATAGTGAGTACCTTCAG-3’ |
| **Primers used for yeast (Y2H) assay** | |
| *GmUBC9* | 5’-GGAGGCCAGTGAATTCATGACGCTTGGCTCT-3’ |
|  | 5’-CACCCGGGTGGAATTCCTAATAGTGAGTACC-3’ |
| *AtHUB2* | 5’-ATGGAGGCCGAATTCATGGAGAATCAGGAA-3’ |
|  | 5’-GATCCCCGGGAATTCTTACATTTTGACAAG-3’ |
| *GmHUB2* | 5’-ATGGAGGCCGAATTCATGGAAAACTCAGA-3’ |
|  | 5’-GATCCCCGGGAATTCTCATATTTTTACAA-3’ |
| **Primers used for BiFC** | |
| *GmUBC9* | 5’-GGAGGCCAGTGAATTCATGACGCTTGGCTCT-3’ |
|  | 5’-CACCCGGGTGGAATTCATAGTGAGTACCTTCAG-3’ |
| *AtHUB2* | 5’-ATGGAGGCCGAATTCATGGAGAATCAGGAA-3’ |
|  | 5’-GATCCCCGGGAATTCCATTTTGACAAGCC-3’ |
| *GmHUB2* | 5’-ATGGAGGCCGAATTCATGGAAAACTCAGA-3’ |
|  | 5’-GATCCCCGGGAATTCTATTTTTACAAAGC-3’ |
