# Supplementary material for: Overexpression of GmUBC9 Gene Enhances Plant Drought Resistance and Affects Flowering Time via Histone H2B Monoubiquitination
Source: Front Plant Sci. 2020 Sep 4;11:555794. doi: 10.3389/fpls.2020.555794 (PMC7498670; doi:10.3389/fpls.2020.555794)
Supplement: Table S5 — The length of the 20 chromosomes of soybean (cultivar Williams 82). [file Table_5.docx]

**TABLE S5 |** The length of soybean (cultivar Williams 82) 20 chromosomes.

| [**Chromosome**](javascript:;) **Number** | [**Chromosome**](javascript:;) **Length (Mb)** |
| --- | --- |
| Chr01 | 56.831624 |
| Chr02 | 48.577505 |
| Chr03 | 45.779781 |
| Chr04 | 52.389146 |
| Chr05 | 42.234498 |
| Chr06 | 51.416486 |
| Chr07 | 44.630646 |
| Chr08 | 47.837940 |
| Chr09 | 50.189764 |
| Chr10 | 51.566898 |
| Chr11 | 34.766867 |
| Chr12 | 40.091314 |
| Chr13 | 45.874162 |
| Chr14 | 49.042192 |
| Chr15 | 51.756343 |
| Chr16 | 37.887014 |
| Chr17 | 41.641366 |
| Chr18 | 58.018742 |
| Chr19 | 50.746916 |
| Chr20 | 47.904181 |
