# Supplementary material for: Overexpression of GmUBC9 Gene Enhances Plant Drought Resistance and Affects Flowering Time via Histone H2B Monoubiquitination
Source: Front Plant Sci. 2020 Sep 4;11:555794. doi: 10.3389/fpls.2020.555794 (PMC7498670; doi:10.3389/fpls.2020.555794)
Supplement: Table S6 — Estimated time of the WGD/segmental duplication events among soybean GmUBC genes. [file Table_6.docx]

############### Parameters ###############

# MATCH_SCORE: 2

# MATCH_SIZE: 1

# GAP_PENALTY: 10

# OVERLAP_WINDOW: 10

# E_VALUE: 100

# MAX GAPS: 100

############### Statistics ###############

# Number of collinear genes: 33, Percentage: 36.26

# Number of all genes: 91

##########################################

## Alignment 0: score=4.0 e_value=2 N=2 Chr01&Chr09 plus

0- 0: GmUBC1 GmUBC36 3e-14

0- 1: GmUBC2 GmUBC37 6e-05

## Alignment 1: score=4.0 e_value=2 N=2 Chr01&Chr17 plus

1- 0: GmUBC1 GmUBC76 4e-14

1- 1: GmUBC2 GmUBC77 6e-06

## Alignment 2: score=14.0 e_value=2 N=2 Chr02&Chr03 plus

2- 0: GmUBC3 GmUBC7 5e-32

2- 1: GmUBC5 GmUBC8 6e-20

## Alignment 3: score=34.0 e_value=2 N=2 Chr02&Chr05 plus

3- 0: GmUBC3 GmUBC14 1e-113

3- 1: GmUBC5 GmUBC18 2e-27

## Alignment 4: score=14.0 e_value=2 N=2 Chr02&Chr07 plus

4- 0: GmUBC3 GmUBC24 2e-17

4- 1: GmUBC5 GmUBC26 7e-28

## Alignment 5: score=14.0 e_value=2 N=2 Chr02&Chr08 plus

5- 0: GmUBC3 GmUBC30 9e-26

5- 1: GmUBC5 GmUBC31 2e-26

## Alignment 6: score=14.0 e_value=2 N=2 Chr02&Chr09 plus

6- 0: GmUBC3 GmUBC36 2e-14

6- 1: GmUBC5 GmUBC37 4e-27

## Alignment 7: score=24.0 e_value=2 N=2 Chr02&Chr12 plus

7- 0: GmUBC3 GmUBC49 6e-44

7- 1: GmUBC5 GmUBC52 2

## Alignment 8: score=24.0 e_value=2 N=2 Chr02&Chr15 plus

8- 0: GmUBC3 GmUBC66 9e-37

8- 1: GmUBC5 GmUBC69 5e-22

## Alignment 9: score=14.0 e_value=2 N=2 Chr02&Chr17 plus

9- 0: GmUBC3 GmUBC76 2e-14

9- 1: GmUBC5 GmUBC77 1

## Alignment 10: score=14.0 e_value=2 N=2 Chr02&Chr19 plus

10- 0: GmUBC3 GmUBC85 1e-32

10- 1: GmUBC5 GmUBC87 2e-25

## Alignment 11: score=14.0 e_value=2 N=2 Chr02&Chr20 plus

11- 0: GmUBC3 GmUBC89 2e-28

11- 1: GmUBC5 GmUBC90 4

## Alignment 12: score=4.0 e_value=2 N=2 Chr03&Chr07 plus

12- 0: GmUBC7 GmUBC24 5e-07

12- 1: GmUBC8 GmUBC25 0.002

## Alignment 13: score=4.0 e_value=2 N=2 Chr03&Chr16 plus

13- 0: GmUBC7 GmUBC70 5e-07

13- 1: GmUBC8 GmUBC71 0.002

## Alignment 14: score=44.0 e_value=2 N=2 Chr07&Chr11 plus

14- 0: GmUBC24 GmUBC44 3e-08

14- 1: GmUBC29 GmUBC45 1e-16

## Alignment 15: score=4.0 e_value=2 N=2 Chr09&Chr11 minus

15- 0: GmUBC36 GmUBC45 1e-16

15- 1: GmUBC37 GmUBC44 6e-05

## Alignment 16: score=24.0 e_value=2 N=2 Chr10&Chr11 plus

16- 0: GmUBC40 GmUBC44 0.01

16- 1: GmUBC43 GmUBC45 0.0001

## Alignment 17: score=4.0 e_value=2 N=2 Chr11&Chr17 minus

17- 0: GmUBC44 GmUBC77 6e-06

17- 1: GmUBC45 GmUBC76 9e-17

## Alignment 18: score=14.0 e_value=2 N=2 Chr16&Chr17 plus

18- 0: GmUBC70 GmUBC76 9e-37

18- 1: GmUBC72 GmUBC77 2e-05
