# Supplementary material for: Overexpression of GmUBC9 Gene Enhances Plant Drought Resistance and Affects Flowering Time via Histone H2B Monoubiquitination
Source: Front Plant Sci. 2020 Sep 4;11:555794. doi: 10.3389/fpls.2020.555794 (PMC7498670; doi:10.3389/fpls.2020.555794)
Supplement: Table S7 — Graphical sequence logo of 91 GmUBCs. [file Table_7.docx]

**DISCOVERED MOTIFS**
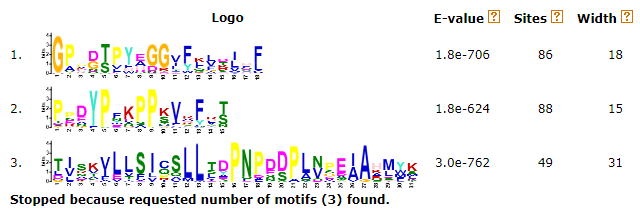


**MOTIF LOCATIONS**
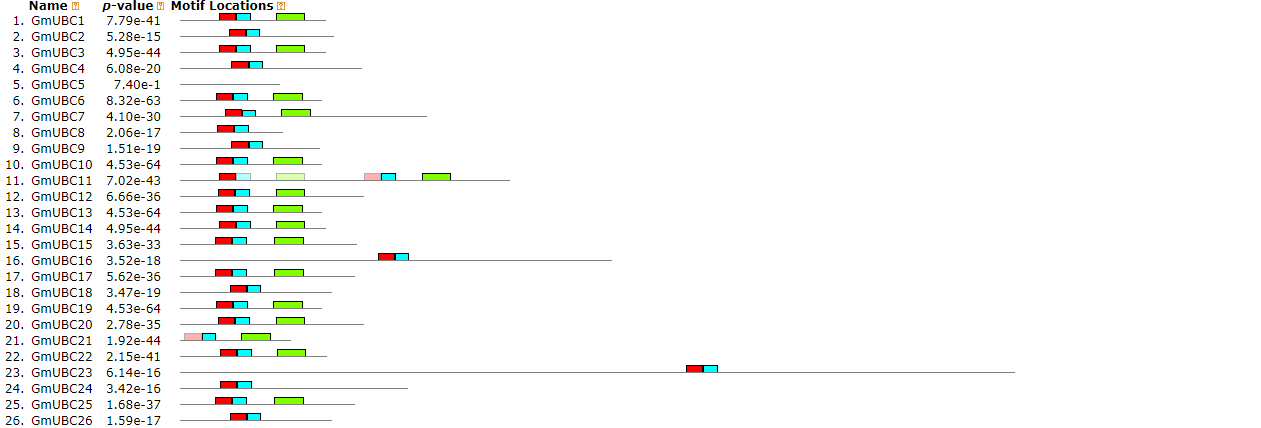

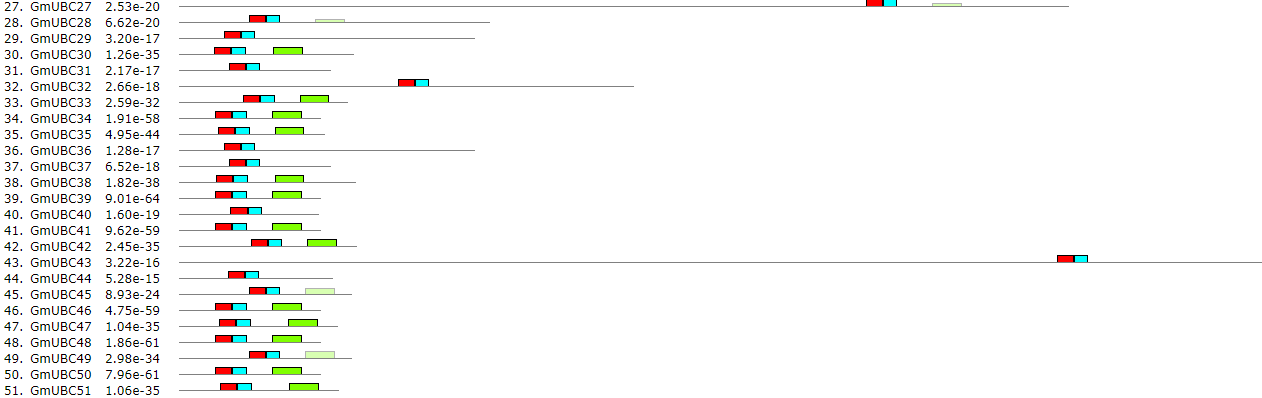

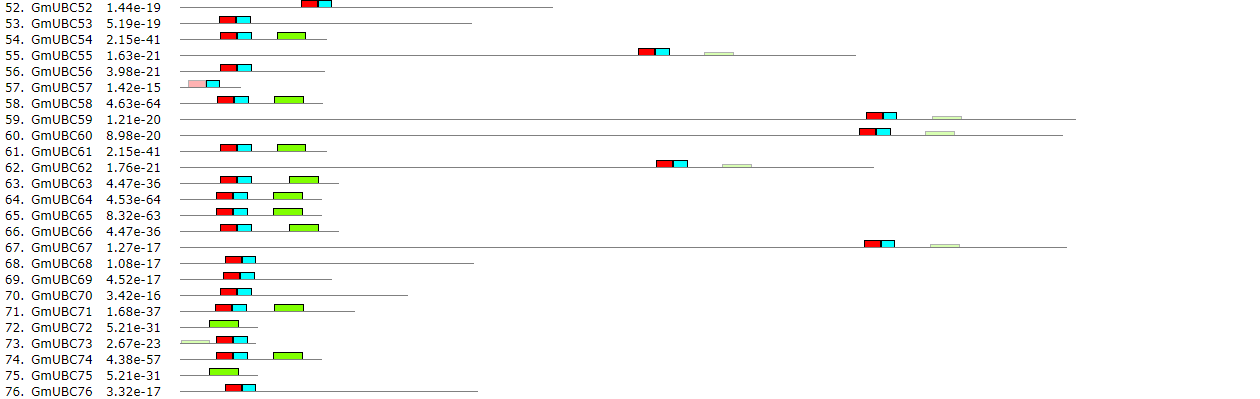


#####
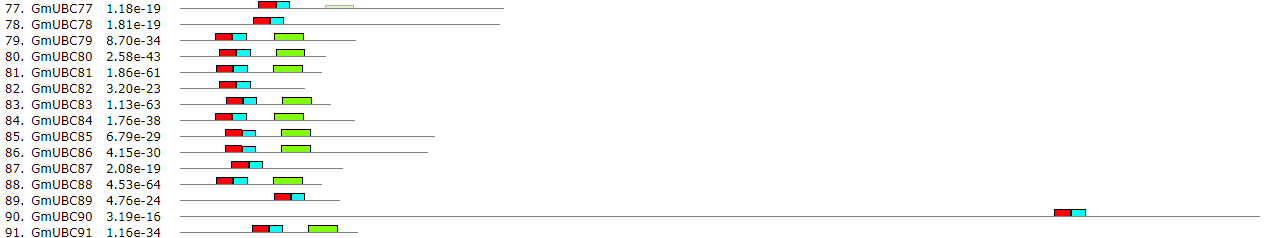

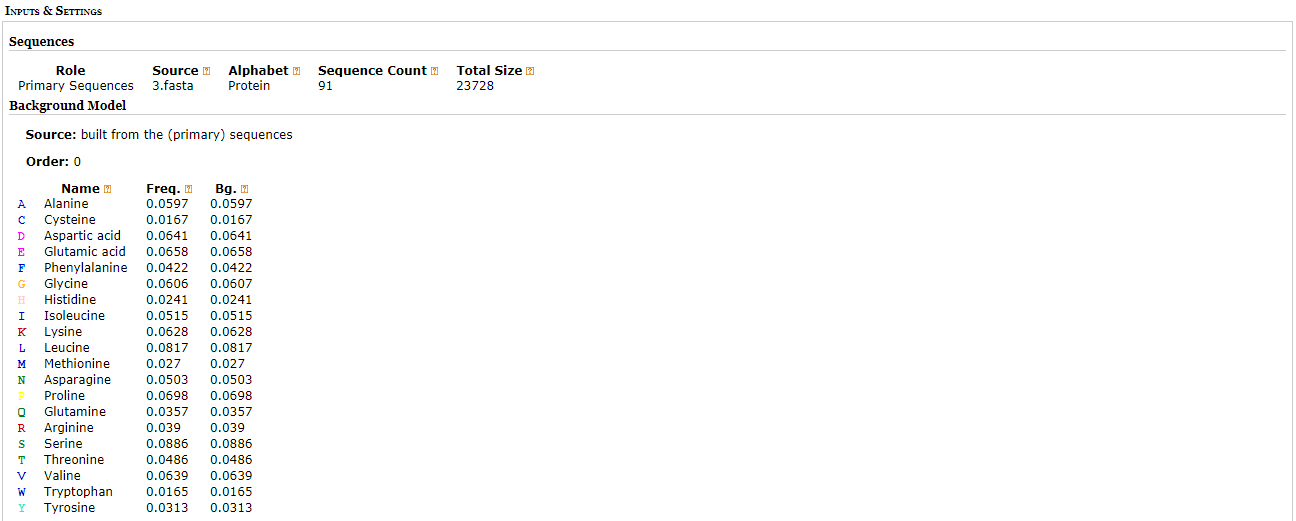

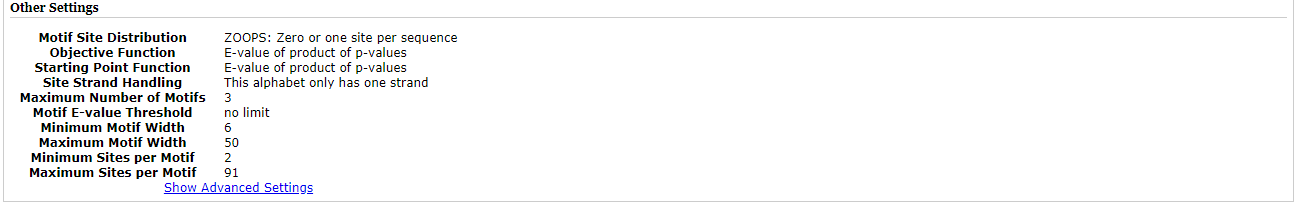
MEME version

5.1.1 (Release date: Wed Jan 29 15:00:42 2020 -0800)

##### Reference

Timothy L. Bailey and Charles Elkan, "Fitting a mixture model by expectation maximization to discover motifs in biopolymers", Proceedings of the Second International Conference on Intelligent Systems for Molecular Biology, pp. 28-36, AAAI Press, Menlo Park, California, 1994.
