# Supplementary material for: Overexpression of GmUBC9 Gene Enhances Plant Drought Resistance and Affects Flowering Time via Histone H2B Monoubiquitination
Source: Front Plant Sci. 2020 Sep 4;11:555794. doi: 10.3389/fpls.2020.555794 (PMC7498670; doi:10.3389/fpls.2020.555794)
Supplement: Table S8 — The total 39 genes related to GmUBC9 in Pearson Correlated Expression analysis. [file Table_8.docx]

**Pearson Correlated Expression**

There are 39   genes with correlated expression > 0.85

| Gene | Defline | Correlation |
| --- | --- | --- |
| [Glyma.02G068000](https://phytozome.jgi.doe.gov/phytomine/report.do?id=99391095) | (1 of 18) KOG2922 - Uncharacterized conserved protein | 0.94307 |
| [Glyma.06G075200](https://phytozome.jgi.doe.gov/phytomine/report.do?id=99507233) | (1 of 30) PF06749 - Protein of unknown function (DUF1218) (DUF1218) | 0.91538 |
| [Glyma.08G120000](https://phytozome.jgi.doe.gov/phytomine/report.do?id=101654266) |  | 0.90875 |
| [Glyma.17G147000](https://phytozome.jgi.doe.gov/phytomine/report.do?id=100177497) |  | 0.90556 |
| [Glyma.20G054900](https://phytozome.jgi.doe.gov/phytomine/report.do?id=99969883) | (1 of 2) PTHR33144:SF3 - PLANT TRANSPOSASE (PTTA/EN/SPM FAMILY) | 0.90512 |
| [Glyma.09G250300](https://phytozome.jgi.doe.gov/phytomine/report.do?id=101207985) | (1 of 4) 4.1.1.3 - Oxaloacetate decarboxylase / Oxaloacetate carboxy-lyase | 0.90185 |
| [Glyma.01G154400](https://phytozome.jgi.doe.gov/phytomine/report.do?id=99540139) | (1 of 5) PTHR19241:SF213 - ABC TRANSPORTER G FAMILY MEMBER 14 | 0.89958 |
| [Glyma.13G306700](https://phytozome.jgi.doe.gov/phytomine/report.do?id=99425102) | (1 of 2) PTHR34126:SF1 - PEROXISOME BIOGENESIS PROTEIN 22 | 0.89856 |
| [Glyma.07G119100](https://phytozome.jgi.doe.gov/phytomine/report.do?id=99571449) | (1 of 6) PTHR15615:SF28 - CYCLIN-U4-1 | 0.88707 |
| [Glyma.18G022500](https://phytozome.jgi.doe.gov/phytomine/report.do?id=99906684) | (1 of 5) K15296 - alpha-soluble NSF attachment protein (NAPA, SNAPA, SEC17) | 0.88152 |
| [Glyma.01G149300](https://phytozome.jgi.doe.gov/phytomine/report.do?id=99519327) | (1 of 2) PTHR10108:SF844 - METHYLTRANSFERASE PMT21-RELATED | 0.88149 |
| [Glyma.11G248300](https://phytozome.jgi.doe.gov/phytomine/report.do?id=99671934) | (1 of 5) K09775 - hypothetical protein (K09775) | 0.8795 |
| [Glyma.03G199500](https://phytozome.jgi.doe.gov/phytomine/report.do?id=100222453) |  | 0.87909 |
| [Glyma.15G188300](https://phytozome.jgi.doe.gov/phytomine/report.do?id=99389023) | (1 of 2) PTHR22970:SF21 - AT-RICH INTERACTIVE DOMAIN-CONTAINING PROTEIN 4 | 0.87795 |
| [Glyma.10G206200](https://phytozome.jgi.doe.gov/phytomine/report.do?id=99753760) |  | 0.87612 |
| [Glyma.14G023700](https://phytozome.jgi.doe.gov/phytomine/report.do?id=99425304) | (1 of 2) K13719 - ubiquitin thioesterase OTU1 (OTU1, YOD1) | 0.87495 |
| [Glyma.14G080900](https://phytozome.jgi.doe.gov/phytomine/report.do?id=99613787) | (1 of 15) PF00685 - Sulfotransferase domain (Sulfotransfer_1) | 0.87035 |
| [Glyma.10G165500](https://phytozome.jgi.doe.gov/phytomine/report.do?id=99398969) | (1 of 4) PTHR22884//PTHR22884:SF376 - SET DOMAIN PROTEINS // SUBFAMILY NOT NAMED | 0.87006 |
| [Glyma.02G170900](https://phytozome.jgi.doe.gov/phytomine/report.do?id=99476553) |  | 0.86818 |
| [Glyma.09G143300](https://phytozome.jgi.doe.gov/phytomine/report.do?id=99690106) | (1 of 8) PTHR11802:SF82 - SERINE CARBOXYPEPTIDASE-LIKE 45-RELATED | 0.86554 |
| [Glyma.09G182500](https://phytozome.jgi.doe.gov/phytomine/report.do?id=100659282) | (1 of 5) PTHR31042:SF16 - CORE-2/I-BRANCHING BETA-1,6-N-ACETYLGLUCOSAMINYLTRANSFERASE FAMILY PROTEIN | 0.86507 |
| [Glyma.20G236700](https://phytozome.jgi.doe.gov/phytomine/report.do?id=99407802) | (1 of 4) PTHR22937//PTHR22937:SF37 - RING FINGER CONTAINING PROTEIN // SUBFAMILY NOT NAMED | 0.86438 |
| [Glyma.13G335200](https://phytozome.jgi.doe.gov/phytomine/report.do?id=100383726) | (1 of 79) PF02701 - Dof domain, zinc finger (zf-Dof) | 0.86432 |
| [Glyma.01G201100](https://phytozome.jgi.doe.gov/phytomine/report.do?id=99437166) | (1 of 2) PTHR12050//PTHR12050:SF4 - LEPTIN RECEPTOR-RELATED // SUBFAMILY NOT NAMED | 0.86375 |
| [Glyma.10G206100](https://phytozome.jgi.doe.gov/phytomine/report.do?id=99444461) | (1 of 4) K12736 - peptidylprolyl isomerase domain and WD repeat-containing protein 1 [EC:5.2.1.8] (PPWD1) | 0.86232 |
| [Glyma.19G255000](https://phytozome.jgi.doe.gov/phytomine/report.do?id=99969674) | (1 of 4) K17525 - chitinase domain-containing protein 1 (CHID1) | 0.86219 |
| [Glyma.08G331800](https://phytozome.jgi.doe.gov/phytomine/report.do?id=99792154) | (1 of 11) PTHR23324//PTHR23324:SF79 - SEC14 RELATED PROTEIN // SUBFAMILY NOT NAMED | 0.86164 |
| [Glyma.13G040600](https://phytozome.jgi.doe.gov/phytomine/report.do?id=99424289) | (1 of 3) PF06017 - Unconventional myosin tail, actin- and lipid-binding (Myosin_TH1) | 0.86138 |
| [Glyma.07G138800](https://phytozome.jgi.doe.gov/phytomine/report.do?id=99608056) | (1 of 19) PF04759 - Protein of unknown function, DUF617 (DUF617) | 0.86129 |
| [Glyma.20G089700](https://phytozome.jgi.doe.gov/phytomine/report.do?id=99618081) | (1 of 4) PTHR23423:SF17 - PROTEIN F40E10.6 | 0.85829 |
| [Glyma.11G090200](https://phytozome.jgi.doe.gov/phytomine/report.do?id=99651901) | (1 of 5) PTHR19241:SF213 - ABC TRANSPORTER G FAMILY MEMBER 14 | 0.85776 |
| [Glyma.11G127900](https://phytozome.jgi.doe.gov/phytomine/report.do?id=99399371) | (1 of 1) PF15024 - Glycosyltransferase family 18 (Glyco_transf_18) | 0.85772 |
| [Glyma.14G048800](https://phytozome.jgi.doe.gov/phytomine/report.do?id=99756776) | (1 of 2) K18467 - vacuolar protein sorting-associated protein 29 (VPS29) | 0.85688 |
| [Glyma.04G090300](https://phytozome.jgi.doe.gov/phytomine/report.do?id=99432009) | (1 of 7) PTHR23029:SF40 - PHOSPHOGLYCERATE MUTASE-LIKE PROTEIN | 0.85598 |
| [Glyma.02G004500](https://phytozome.jgi.doe.gov/phytomine/report.do?id=100465576) |  | 0.85595 |
| [Glyma.20G166800](https://phytozome.jgi.doe.gov/phytomine/report.do?id=100786551) | (1 of 4) PTHR21654:SF9 - TRIHELIX TRANSCRIPTION FACTOR GT-2 | 0.85396 |
| [Glyma.15G122500](https://phytozome.jgi.doe.gov/phytomine/report.do?id=100158841) |  | 0.85369 |
| [Glyma.04G089500](https://phytozome.jgi.doe.gov/phytomine/report.do?id=100340200) |  | 0.85178 |
| [Glyma.U004300](https://phytozome.jgi.doe.gov/phytomine/report.do?id=100661548) | (1 of 4) PTHR22904//PTHR22904:SF334 - TPR REPEAT CONTAINING PROTEIN // SUBFAMILY NOT NAMED | 0.85112 |
